# Advances in Plasmonic Sensing at the NIR—A Review

**DOI:** 10.3390/s21062111

**Published:** 2021-03-17

**Authors:** Paulo S. S. dos Santos, José M. M. M. de Almeida, Isabel Pastoriza-Santos, Luís C. C. Coelho

**Affiliations:** 1 INESC TEC—Institute for Systems and Computer Engineering, Technology and Science, and Faculty of Sciences, University of Porto, Rua do Campo Alegre, 4169-007 Porto, Portugal; paulo.s.santos@inesctec.pt; 2 Faculty of Engineering, University of Porto, Rua Dr. Roberto Frias, 4200-465 Porto, Portugal; 3 Department of Physics, School of Science and Technology, University of Trás-os-Montes e Alto Douro, 5001-801 Vila Real, Portugal; jmmma@utad.pt; 4 CINBIO, Universidade de Vigo, Campus Universitario Lagoas, Marcosende, 36310 Vigo, Spain; pastoriza@uvigo.es; 5 SERGAS-UVIGO, Galicia Sur Health Research Institute (IIS Galicia Sur), 36312 Vigo, Spain

**Keywords:** localized surface plasmon resonance, LSPR, SPR, near infra-red, NIR, optical fiber sensors, nanoparticles, plasmonics

## Abstract

Surface plasmon resonance (SPR) and localized surface plasmon resonance (LSPR) are among the most common and powerful label-free refractive index-based biosensing techniques available nowadays. Focusing on LSPR sensors, their performance is highly dependent on the size, shape, and nature of the nanomaterial employed. Indeed, the tailoring of those parameters allows the development of LSPR sensors with a tunable wavelength range between the ultra-violet (UV) and near infra-red (NIR). Furthermore, dealing with LSPR along optical fiber technology, with their low attenuation coefficients at NIR, allow for the possibility to create ultra-sensitive and long-range sensing networks to be deployed in a variety of both biological and chemical sensors. This work provides a detailed review of the key science underpinning such systems as well as recent progress in the development of several LSPR-based biosensors in the NIR wavelengths, including an overview of the LSPR phenomena along recent developments in the field of nanomaterials and nanostructure development towards NIR sensing. The review ends with a consideration of key advances in terms of nanostructure characteristics for LSPR sensing and prospects for future research and advances in this field.

## 1. Introduction

Recent nanomaterials and nanostructure developments have produced enormous improvements in the field of nanoplasmonic sensing, enabling an increase of refractometric sensitivity alongside a better control over plasmonic wavelength tunability. This is one of the reasons behind the growth in relevance over the last decades for both biological and chemical sensing, reaching many scientific fields, from medical diagnostics and drug delivery to food quality/safety and environmental monitoring [1]. Among the available technological platforms to interrogate these sensing devices, optical fiber solutions benefit from electromagnetic (EM) interference immunity, potential low-cost, mechanical flexibility, and low attenuation factors in the near infra-red (NIR), specially at C and L telecommunications bands (1530–1625 nm). The nanoplasmonic material could be used to functionalize optical fiber gratings, such as long-period fiber gratings (LPFGs) and fiber Bragg gratings (FBGs), or other nanostructures, such as etched or tapered fiber sections, to achieve high performance bio and chemical sensing [2,3]. These mechanisms allow to retrieve the light travelling on the fiber core, by total internal reflection, to the cladding where an evanescent wave is created and capable of interaction with the external medium, thus providing a great simplification of the coupling conditions. Moreover, there is a current trend towards the creation of sensing networks using optical fiber platforms, thus avoiding the conversion of photonic quantities to an electronic equivalent at each sensing point and thereby potentially reducing the overall costs and increasing sensing flexibility.

Although recent and well cited reviews on localized surface plasmon resonance (LSPR) biosensors can be found in the literature, these have not been focused on LSPR sensing at NIR wavelengths, which we found to be a current trend in the sensing field [4,5,6,7]. More precisely, in the EM spectrum, comprehending the wavelength window between the NIR-II (1100–1350 nm) and NIR-III (1600–1870 nm). Therefore, this aspect will be addressed herein, where we will provide the body of knowledge in this area, reviewing the key fundamental aspects and recent progress made in the development of ultra-sensitive LSPR sensors at NIR that could be used on optical fiber-based sensors. Thus, this report will give an overview of the underlying principles and characteristics, a comparison of several nanoparticle (NP) nanostructures and nanomaterials for both chemical and biological LSPR sensing, ending with future perspectives of research, aiming for potentially plasmonic sensors at the C and L telecommunication bands for optical fiber deployment, thereby benefiting from the current mature and low-cost status of optical fiber technology.

### 1.1. Localized Surface Plasmon Resonances

The plasmonic information of LSPR is fully contained in the complex dielectric function of the nanomaterial and nanostructure [8,9,10,11]. These properties are well described in the literature thoroughly explaining the principles of LSPR. Therefore, the authors in this will review will strictly discuss the NP parameters towards the LSPR at the NIR regime. For additional theory behind the mechanisms of operation, the authors encourage the reader to explore the articles referenced here whenever a more detailed description over the essentials is needed [5,12,13,14,15,16,17,18,19,20].

Nanospheres (NS) being the simplest nanostructures, present the simpler interactions with the incoming EM field, e.g., originated from a laser source. As described by Ru et al. [8] the response to the EM field on a small metallic sphere (much smaller than the wavelength of the incident beam) results in am EM field E_NS_ given by:

(1) $$E_{NS}=\frac{3\varepsilon_{M}}{\varepsilon +2\varepsilon_{M}}E_{0}$$

where *E*_0_ is the incoming EM field,${\;\varepsilon }_{M}$ is the relative dielectric constant of surrounding medium and ϵ the complex metal dielectric function ($\varepsilon \;=\varepsilon^{\prime }+i\;\varepsilon^{\prime \prime }$). The resonance condition is achieved when the denominator gets to zero, i.e., when:

(2) $$\varepsilon =-2\varepsilon_{M}$$

Naturally, in practice when this condition is met, the field inside the sphere will not rise to infinite due to several loss mechanisms. For standard dielectric materials ε ranges from 1 to 10. Therefore, if equation 2 is to be met, NP must have a small $\varepsilon^{\prime \prime }$, and $\varepsilon^{\prime }=\;-2\varepsilon_{M}$. Since any change in $\varepsilon_{M}$, will change the resonant wavelength, thus creating a refractometric sensor. This condition imposes that the resonance wavelength is only dependent on the nanomaterial, while not being entirely true, since NP size also affects the resonant condition, does not allow to easily shift this nanostructure towards greater wavelengths as happens to other nanostructures. As a comparison, nanorods (NR) allow the tuning through NP elongation adjustments, as presented by Equation (3).

(3) $$E_{ellipsoid}=\frac{\varepsilon \;-\;\varepsilon_{M}}{3{\;L}_{i}\varepsilon +\left(3-3L_{i}\right)\varepsilon_{M}}E_{0}$$

where *E_ellipsoid_* is the field inside the NP, and $L_{i}$ represents the geometrical factor, which essentially characterizes the ellipsoid curvature along the corresponding axis. In the special case, $L_{1}=L_{2}=L_{3}=\;\frac{1}{3}$ corresponds to the special case of a NS. Now, the resonance condition changed and is dependent on a geometrical variable, as presented in Equation (4).

(4) $$\varepsilon =-\left(\frac{1}{L_{i}}+1\right)\varepsilon_{M}$$

Thus, two equally sized NPs composed of the same nanomaterial, but differing in nanostructure, will have different optical and refractometric properties. As will be seen, in general, a NP elongation (increase of aspect ratio) will redshift the resonance band, along an increase of refractive index (RI) sensitivity [5].

**Figure 1:**
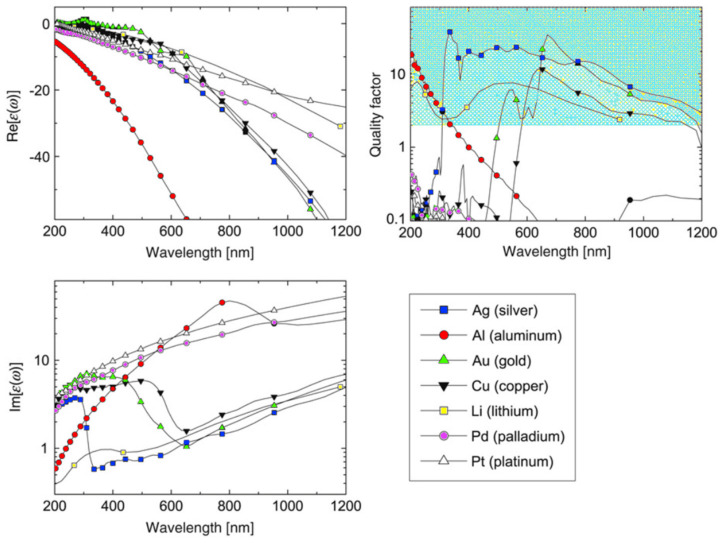
Optical properties of a selection of metals. The real (**top**) and imaginary (**bottom**) parts of ε are plotted against wavelength on the left. Also shown (**top-right**) is the predicted approximate Q-factor of LSPR for a metal/air NP interface. The shaded area is the area of interest to many plasmonics applications. Adapted with permission from [21].

Since plasmonic effects happen when photons interact with a material, exciting free electrons, metals are obvious candidates for plasmonic materials. Moreover, the nanomaterial complex permittivity also provide aid in the LSPR characteristics discussion in terms of resonance width. In general, nanomaterials losses increase with $\varepsilon^{\prime \prime }$, as happens with metals for NIR wavelengths, which can be seen at the bottom section of Figure 1.

In short, $\varepsilon^{\prime }$ is responsible for the matching of plasmonic resonance wavelength, and in general must be small, while $\varepsilon^{\prime \prime }$, leads to absorption losses and consequently to broadening of the plasmonic band, limiting overall performance of the LSPR sensor [22].

Inter-band transitions (bound electrons optically excited to a higher energy band) are the main factor responsible for the $\varepsilon^{\prime \prime }$ increase at NIR wavelengths. Along the metals displayed, this plagues specially aluminum at these higher wavelengths of interest [20]. This means that sharp resonances at the VIS, usually lead to broader bands at the NIR and MIR [23]. To overcome some of these difficulties imposed by the inter-band transitions, several new meta-materials were targeted as potentially high-performance nanomaterials for these applications, thus overcoming the inevitabilities when using noble metals. This can be achieved mainly by doping dielectric NPs, which would otherwise perform very poorly as plasmonic devices, since, by definition, they lack electrons in the conduction band. As will be seen later, doped semiconductors could present an alternative for LSPR at the NIR. This is due to the near absence of inter-band transitions, which in many cases, occur in the UV [8].

The Drude model help to understand better the source of losses. The free-electron response in metals can be described by [24]:

(5) $$\varepsilon =\;\varepsilon^{\prime }+i\;\varepsilon^{\prime \prime }=\varepsilon_{b}-\frac{\omega_{p}^{2}}{\omega^{2}+\gamma^{2}}+i\;\frac{\omega_{p}^{2}\gamma }{\omega^{2}+\gamma^{2}}$$

where $\varepsilon_{b}$ is the polarization response from the core electrons, that ultimately represents the inter-band transitions that coexist with the free-electron contribution modelled by the second term, $\omega_{p}$ is the plasma frequency, and γ is the Drude relaxation rate, corresponding to the collision rate of free electrons with the crystal or impurities. Being responsible for the ohmic losses, they are inversely proportional to the average NP size [25,26,27]. Furthermore, the plasma frequency can be defined as:

(6) $$\omega_{p}=\sqrt{\frac{Nq^{2}}{\varepsilon \times m^{*}}}$$

where *N* is the carrier concentration, typically about $10^{23}$ cm^−3^ for metals, *q* the electron charge, ε the free space permittivity, and $m^{*}$ the effective electron mass. Since $\omega_{p}$ is directly proportional to N, a lower carrier concentration can reduce losses. This can be achieved using semiconductor materials, with typical undoped carrier concentrations for Si, Ge, and Ga of $9.65\times 10^{9}$, $2.33\times 10^{13},$ and $2.1\times 10^{6}$, respectively [24,28,29]. The same effect is obtained if γ is minimized. γ minimization attempts have been successfully found in the literature by exploring more complex nanostructures with un-degenerated symmetries, such as square-particle or nano-antennas [25,30]. Nevertheless, other loss mechanisms in metal films linked to surface and boundary roughness could also be relevant [31,32].

The nanoplasmonics research field, which has been mostly restricted to the use of noble metal NP, nowadays hosts a plethora of studies on new and alternative plasmonic nanomaterials [20,33]. Metamaterials are such an example, generally composed by metals and dielectrics, where the metals are also called the plasmonic elements of metamaterials due to their interaction with light. LSPR has been widely applied in enhancing luminescence [34,35], sensing [36,37,38], photo-catalysis [18,39,40], photo-therapy, and photonic devices, only to mention a few examples [41,42,43,44].

However, LSPR based sensors are known to have an RI sensitivity of at least an order of magnitude lower when compared to an SPR sensor, with sensitivities, typically, not exceeding 100–400 nm/RIU at VIS wavelengths [45]. Nevertheless, their use can prove to be beneficial due to their sensing optimization through variations of the size, shape, interparticle distance, and nanomaterial choice, allowing them to achieve plasmonic resonances throughout the NIR regime [46,47].

Moreover, the research for new materials and nanostructure optimization towards the NIR, where low-cost and long-range sensing capacity can become a reality, when supported by optical fiber technology, presents itself as a promising field to be explored soon. Thus, providing a step towards the affirmation of real world and large-scale implementation of plasmonic sensing devices out of laboratory environments.

### 1.2. Nanoparticle Fabrication

Since standard synthesis protocols for NP appeared in the literature some decades ago, one can get the impression that NPs of any material, size, and shape are easily synthesized. Even for well-known NP, this impression is not always correct. Therefore, a need for a set of NP synthesis methods targeting LSPR at NIR wavelengths has been identified. More precisely, in recent years a world-wide effort has been observed in the development of newer and better fabrication techniques. Among these, the most important are chemical/electrochemical synthesis, electron-beam lithography (EBL), nanosphere lithography (NSL), and colloidal assembly, the latter being the most widely applied synthesis method to date, and in particular the seeding methodology, mainly due to its simplicity, high yield, ease of size control, fabrication speed, efficiency, structural flexibility, post-synthesis functionalization ease, and colloidal stability [48,49,50].

Briefly, a typical seed-mediated growth process involves two steps, synthesis of seed NP and their subsequent development in growth solutions containing metal precursors, reducing and shape-directing reagents.

Typically, the process to fabricate Au NPs starts with the boiling of gold salt (HAuCl_4_), which will be reduced by a reducing agent, such as, sodium citrate (Na_3_C_6_H_5_O_7_) in a dilute solution. Upon dissociation, the citrate ions (C_6_H_5_O_7_^3−^) reduce Au^3+^ to Au, which aggregates into seed crystals, and posteriorly grows to form Au NP. The seed growth step could be made to impose isotropic or anisotropic growth, e.g., by adding a surfactant to cap certain crystal faces and promoting growth only in selected directions. This method corresponds to the, well-known, Frens method [51]. Citrate ions reduce gold and act as capping agents to stabilize the resulting gold colloid (12–50 nm) by electrical repulsion forces [52]. The use of stronger reducing agents, such as sodium borohydride, is not of interest for NIR plasmonics since it only produces smaller NPs (2–10 nm).

The reducing agents are responsible for the negative effect of the Au NP capped with weakly adsorbed ions and thus tending to aggregate upon stresses caused by centrifugation, dilution, dialysis, and upon contact with biological mater. Post-synthesis surface modification with small stabilizing molecules (e.g., thiolated molecules, amino acids, or proteins) or polymers (e.g., poly-vinyl alcohol, polyethylene glycol, and poly-electrolyte) is essential to avoid aggregation and improve colloidal stability [53].

Furthermore, this method can produce a wide variety of nanostructures exhibiting pronounced redshifts on the LSPR band towards the NIR, among these nanostructures of interest lie the elongated ellipsoid like nanostructures as nanorice, nanocarrots and the famous NRs, as well as, nanotriangles, nanocubes, nanoshells, and nanorings [54,55,56,57].

Among these nanostructures enabling NIR plasmonics, NRs have attracted much interest because of the plasmonic tuning capabilities obtained from the aspect ratio control. Typically, AgNO_3_ is used for shape induction, cetyltrimethylammonium bromide (CTAB) as the template and ascorbic acid (AA), as the reducing agent. Noting that a slight cytotoxicity is created by the CTAB excess, enveloping the NR surfaces, which is not relevant for biosensing applications, as it in the field of medical applications.

Currently there are great works explaining in detail this method, and we will only point the reader to them, and here we will briefly make some references to not pivot the goal of this review [27]. Some of the latest methods that contributed to achieve better NRs characteristics aiming NIR wavelength operation focused on higher aspect ratios.

In 2009, Altansukh et al. [58] produced Au NR in highly acid environments (pH between 2.2 and 3.3) in the presence of CTAB, culminating in a maximum aspect ratio of 18.7. This work produced higher aspect ratios, but as in the work reported by Jana et al. [59], the yield was still a major drawback. Later in 2011, Caseri et al. [60] kept pursuing high aspect ratio Au NRs, with better reproducibility and higher yields, reporting an LSPR band located at a maximum of 1955 nm.

In the same work, a linear dependency on LSPR band was presented with an aspect ratio increase of λ= 115.3 AR + 387.6, where AR represents the aspect ratio. The proportional constant in this relation, nevertheless, varies from other constants presented in literature for Au NR, as much as nearly 20%. Such examples are the work Link et al. [61], presenting λ= 103.7 AR + 392.1, or that presented by Brioude et al. [62]. These works suggest that, for the optical C or L telecommunications band, an aspect ratio of 10 would suffice, which can be, achievable with arguable ease. Wang et al. [63] reported NRs with incredible high aspect ratios up to 85.5, produced via Seed-mediated growth, which could be used to place the longitudinal LSPR mode from 1500 nm to 4450 nm, as reported.

The seed-growth mediated method is also responsible for other NIR nanostructures and nanomaterials, as presented by Wu et al. [64] with a report of Ag nanotriangles operating at NIR wavelengths. Other nanotriangles shown potential for NIR LSPR sensing, as those produced by lithographic means [65,66,67].

Beyond purely colloidal methods, lie the relevant lithographic field, which can enable achieve nanostructures fabrication by “top-down” or “bottom-up” approaches. The top-down route starts the development by the macro structural design, usually involving lithographic patterning techniques using short-wavelength optical sources, to build the nanostructures in place, so that no assembly step is needed. The bottom-up approach starts the construction from the atomic or nanoparticle level, which will then self-assemble into larger and more complex nanostructures. Additionally, due to its nature, it is usually capable of achieving higher resolutions.

Electron beam lithography (EBL) belongs to the bottom-up lithographic technique family, consisting of the scan of a focused electron beam to create patterns on an electron-sensitive film (resist), enabling sub 10 nm nanostructures, as nanowires or nanodots. This feature sizes are impossible to other top-down approaches, as the photo-lithography technique, whose diffraction imposes resolution limits [68,69]. Nano-imprint lithography (NIL) can achieve around the same resolutions as EBL but simpler and at a potentially lower cost, creating patterns by mechanical deformation on the resist. This technique allows the creation of nanostructures supporting LSPR at NIR, such as the metal-insulator-metal (MIM) nanodisks presented by Wei et al. [70] or Chang et al. [71], which led to huge sensitivities around 1500 nm/RIU. Moreover, currently in the literature there exists an enormous range of publications using the NIL technique to achieve a myriad of MIM nanostructures supporting NIR LSPR [72,73].

New hybrid methods, i.e., including characteristics from both bottom-up and top-down methods, are available, such as the notable case of NSL, also known as colloidal lithography, which promises inexpensive fabrication. This method consists of NS suspended in colloidal solution, followed by their deposition on a substrate, that will cause them to self-assemble into a hexagonal-close-packed (HCP) nanostructure. Then, the nanomaterial that will form the nanostructure of interest is deposited on top of the HCP assembly, typically done by physical vapor deposition (PVD). At this moment, if a tilt is applied to the substrate, geometries as nanocrescents could be developed, ending with a lift-off step [74]. It is important to mention that the thin film thickness deposited directly impacts the nanoring thickness, and consequently the LSPR wavelength. Larsson et al. [23] reproduced these nanorings following the described method and achieved the manufacture of a LSPR biosensor spanning wavelengths from 550 to 1800 nm, along a high RI sensitivity around of 880 nm/RIU. Toma et al. [75] has also shown these nanorings at NIR wavelengths from 1100 to 1600 nm with RI sensitivities between 805 and 1020 nm/RIU.

Nanorings obtained through colloidal lithography, are known to achieve LSPR at NIR, as presented in works, such as that of Alzpurua et al. [74] who described a route towards synthesis for LSPR at the NIR. For such nanostructures, the typical tuning parameter is the thickness to radius ratio. More precisely, causing the LSPR band to move towards higher wavelengths upon a reduction on the thickness to radius ratio.

Other geometries, e.g., nanocrosses, are nonetheless usually obtained by lithography means, and are amongst the highest sensitive nanostructures available at NIR wavelengths. A great example of such structure was presented by Fortuño et al. [76] with a gold nanocross array produced by EBL. In general, this process, offers a very precise control over the nanostructure dimensions, thus providing great means to achieve high sensitivities, especially when aided by interparticle coupling enhancements as happens with nanoarrays and nanoslits.

Galvanic replacement is another method that could be followed to produced bimetallic or even hollow NPs with thin walls, typically using a combination of Au and Ag. The galvanic replacement reaction was studied extensively and can be read in articles such as the one presented by da Silva et al. [77]. Among the available methods that encompassed by galvanic replacement, the ones including anisotropic growth are of special interest for LSPR at NIR wavelengths [78].

In such articles one can find several exotic nanostructures, such as nanodendrites, nanoflowers, tadpoles, nanowires, nanobipyramids, nanoboxes, or nanorattles [79]. Although the immense choice for nanostructures that could present very interesting spectral features, their parameters are usually poorly defined causing an immense variability, low uniformity, and difficulties for large-scale production. Nevertheless, progresses in this field are expected, since a deeper understanding of the NPs nucleation process will allow for better control over the nanostructure parameters.

In conclusion, the tremendous progress reported in NP chemistry has produced rigorous methodologies to control the size, shape, and nanostructure surface, allowing for the immense variety of nanostructures that will be discussed next, and thus enabling a wide range of choices in terms of RI sensitivity and spectral characteristics to pursue LSPR at the NIR using optical fiber technology as a plasmonic platform. Colloidal methodologies are identified as being of special interest to achieve large-scale production with a high yield at lower costs, and with a relatively simple setup, especially for the stable Au NR. These NPs, made by chemical reactions in solutions, or self-assembled into nanostructures can therefore be further deposited onto optical fibers and functionalized to achieve sensing of specific analytes.

### 1.3. Optical Fiber Functionalization and Sensing Applications

Nanoparticle functionalization of an optical sensing probe is a preponderant theme for sensing applications since one must be careful with some characteristics. Indeed, selectivity, signal to noise ratio (SNR), linearity, sensitivity, compatibility, and stability, are especially in environmental sensing. The functionalization is the key component responsible for the interaction with the environment, with spectral dynamics caused by analyte binding, inducing RI changes.

An NP-based optical fiber sensor functionalization can be considered a two-step process, namely the adhesion of NPs to the fiber sensor and deposition of organic layers on the NPs surface to gain specificity to the analytes of interest. The NPs surface modification is achieved through the adhesion of specific organic layers, such as, monomers and polymers or inorganic layers such as metals or oxides. This process involves the conjugation of molecules onto the NP surface through non-covalent or covalent binding, amorphous NP coating, or even surface epitaxial growth.

Among the inorganic layers, gold and silica are the most used materials used for NP coating, since they provide high stability and a solid path for posterior functionalization. However, one should be careful since doing so can dramatically shift the resonance band away from the region of interest, as a bare NP will present different spectral characteristics than a functionalized one. Additionally, cross sensitivity and reproducibility will inevitably degrade the sensing performance. Another issue is linked to the density of NP on the fiber surface, since higher densities can increase the quantity of analytes immobilized on their surface and consequently have a more pronounced spectral response. This favours the use of arrays or slits and creates a problem with the nanostructures obtained from colloidal techniques, where the nanoparticles are usually dispersed and where aggregation is a problem.

Finally, the integration of these functionalized NPs on a specific optical fiber region responsible to gather the light travelling in its core, e.g., through a LPFG, or a multi-mode section on a single mode fiber, also presents an interesting problem. To date some techniques have been proposed. Smythe et al. [80] presented a transfer method using a pre-polymer material between a PDMS layer and a blank silicon wafer. Then the pre-polymer material is cured with UV light and the components separated from the silicon wafer. Next the polymer-PDMS is pressed again a silicon wafer containing the NPs adsorbing them. Finally, the polymer-PDMS containing the NPs are pressed against an optical fiber facet and the polymer layer is removed by oxygen plasma etching. This method could also be used to deposit the NPs onto a grating on the fiber, or a multi-mode section, but not to a tapered region due to mechanical fragility. Even though it is a multi-step process, it is a straightforward method to place the NPs onto a single-mode optical fiber.

Lin et al. [81] presented a more facile method to get the NPs onto an optical fiber tip, based on EBL. The authors started by a 2 nm Cr deposition on a fiber facet to promote the adhesion of an Au layer. Finally, using EBL patterned the desired nanostructure. This method has the disadvantage of not being able to apply on NPs colloidally produced, as the promising high aspect ratio Au NRs. Additionally, its refractometric capabilities were tested, obtaining sensitivity of 196 nm/RIU. Finally, they functionalized the nanostructure with a monolayer of biotin via a covalent bonding, measuring a streptavidin in DI-water of LOD of 6 pM. Later, Zeng et al. [82] produced a more simple and fast approach, that can be completed in a few seconds, by in situ photo-reduction of Ag+ cations onto Ag0, only requiring a light source to be illuminate the fiber core. This method although producing an ensemble of NPs precisely located at the end-facet core, it is not suitable to create anisotropic NPs. Regarding the sensitivity of this LSPR sensor, a mere 65 nm/RIU was obtained, suggesting that the same method should be improved. Manoharan et al. [83] recently presented an in-situ growth of seed NPs immobilized directly on an optical fiber by immersion on the growth solution. The authors utilized an amine-functionalized U-bent sensing probe (1.0 ± 0.2 mm), and the seed concentration on the fiber surface controlled by the incubation duration, also allowing for real-time monitoring of the spectral features as the NPs synthesis progressed. Finally, RI sensitivity was tested in terms of absorbance per RIU change, resulting in approximately four times increase between the Au NPs functionalized fiber, when compared to the bare sensing-probe.

Between the presented functionalization methods, the bottom-up approaches present a more viable approach to deposit the NPs on non-planar nanostructures as optical fibers, regarding fabrication costs and ease. Additionally, presenting a facile approach for the synthesis via seed-mediated growth techniques of anisotropic NPs, such as NRs, nanostars or bi-pyramids, is ideal for NIR applications.

Now, let us briefly point to some bio-sensing applications, focused mainly on water contaminants, as this makes a perfect example of the advantages of combining optical fiber sensing technology with plasmonic NP-enabled sensing. Namely, we report the LSPR optical fiber sensors enabled through functionalized NPs targeted at a set of analytes. Among the most common and eminent water contaminants nowadays lies the controversial Glyphosate, a popular herbicide, used extensively in large agricultural fields.

Nafisah et al. [84] has recently reported the use of gold bi-pyramids and NRs as LSPR sensors capable of detecting glyphosate concentrations as low as 0.1% (*w/w*) in DI-water. This sensor was based on the direct impact of Glyphosate concentration on RI, therefore lacking any kind functionalization. Nevertheless, the study presented low sensitivities of 55 and 35 nm/RIU for the bi-pyramids and NRs, respectively. Finally, the authors reported lower cross-sensitivity towards chlorpyrifos, acetic acid and acetone.

Regarding functionalization compounds for glyphosate sensing, cysteamine is a common chemical compound biosynthesized in mammals. The work presented by Heidemann et al. [85] is such an example. Here, the authors utilized an LPFG sensor with cysteamine functionalized gold NPs. This sensing system also works by the RI change induced by the reaction of cysteamine with glyphosate molecules. The work presented a LOD of 0.02 μM and a sensitivity of 3.5 nm/μM. This work can also be ported to work at higher wavelengths, through higher order modes, even characterized by higher RI sensitivities. Furthermore, this is an easy way to have a biosensing system at the C and L optical telecommunication bands due to the easiness of LPFG fabrication at these wavelengths with methods such as electric-arc discharges [86].

Organophosphates, originated from agricultural activity, are another concerning family of water contaminants. Dimethoate is an example of these organophosphate insecticides that have been targeted of special relevance. Li et al. [87] presented, last year, a LSPR sensor based on gold NPs for detection of dimethoate, achieving a detection range of 10–100 nM and a LOD of 5.5 nM. The authors took advantage of the NPs aggregation in the presence of NaOH, caused by electrostatic attraction, the same effect occurs in the hydrolyzation of dimethoate, as shown by the authors. Saddani et al. in [88] produced an optical fiber biosensor for mercury detection in the range 0.1 to 540 parts per billion in biological and environmental samples. To do so, Chitosan capped Au NP on bovine serum albumin (BSA)

Inorganic sensing is also possible, as in the case of Mercury, being a diversely bioaccumulating heavy metal pollutant. To sense the presence of such ions, Zhong et al. [89] produced an optical fiber LSPR sensor to detect Hg+ ions through functionalization of gold NPs with chitosan and poly-acrylic acid (PAA) bilayers, resulting in a LOD of 1 μM and a sensitivity of 0.51 nm/ppm. Sadani et al. [88] also reported a LSPR sensor with gold NPs functionalized with bovine serum albumin (BSA), reaching a LOD of 0.1 ppb, below the 2 ppb limited stated by the EPA for drinking water.

### 1.4. LSPR Sensing Parameters

In LSPR sensing, several quantifiers allow to assess the quality of the plasmonic platform as well as the comparison between different LSPR sensors. Next will be defined some figures of merit that are more commonly used to better describe them.

Refractive index sensitivity (S) often measured in nm/RIU is arguably the most important one, and can be defined as:

(7) $$S=\frac{\delta \lambda }{\delta n}$$

represents the ratio between resonance wavelength shift (δλ typically measured in nm) and the RI variation (δn) of the surrounding medium. The figure of merit (FOM) is also considered, being defined as the ratio of the RI sensitivity to the resonance width [90].

(8) $$F\mathrm{OM}=\frac{S}{FWHM}$$

where *FWHM* represents the full width at half maximum of the resonance band in nm.

A third useful parameter to quantify the grade of the LSPR sensor is the Q-factor, being strongly affected by absorption, caused by large $\varepsilon^{\prime \prime }$. Thus, the broader the resonance becomes the smaller the Q-factor. Moreover, the bandwidth can provide a measure of how strong of the resonance can be, and ultimately can be viewed as a degree of the resonance compactness. The Q-factor is expressed as:

(9) $$Q=\frac{\lambda_{central}}{FWHM}$$

where $\lambda_{central}$ is the central wavelength of the plasmon resonance. Ru et al. [8] also presented a Q-factor comparison for the most used metals, as reproduced in Figure 1.

A last parameter is the spectral resolution, typically defined as the minimum detection limit. Unlike the conventional SPR sensors, LSPR have a resolution, which can be improved by the size and geometry of the NP. These conjugated factors can describe the sensing performance and will be used to evaluate various types of sensors throughout this review.

## 2. Nanostructures

### 2.1. Introduction

Nanoparticles are known to be capable of showing highly localized plasmonic resonances when an incident light at a certain wavelength, causes their free electrons to resonate, thus exerting a tearing force, counteracted by Coulomb forces [91,92,93]. Herein, this section will present a set of nanostructures whose resonances are located at NIR wavelengths. Furthermore, their spectral features being dependent on NP geometry and interparticle coupling, their properties will be discussed and compared.

**Figure 2:**
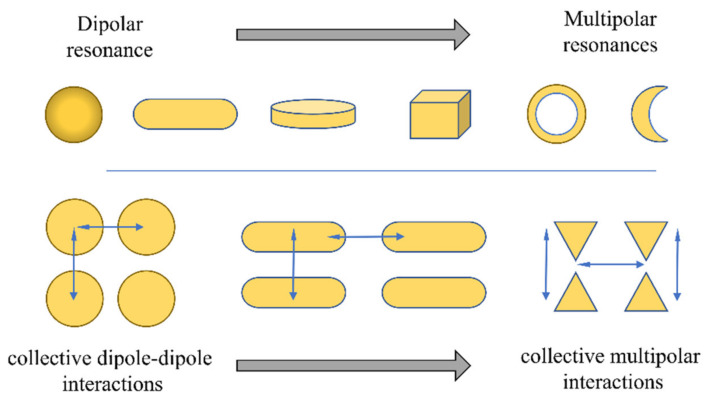
Schematic diagram for several NP nanostructures that are used in LSPR sensors, shifting from dipolar resonances to multipolar ones. These are presented in increasing order of tip sharpness. The lower section represents the interparticle interactions in array nanostructures, that will also cause additional changes on the spectral characteristics.

In recent years, interesting progresses towards highly performant nanostructures have been reported [94]. These nanostructures not only present higher refractometric sensitivities, but also a higher control over their geometries. The latter is of special relevance, since it allows precise plasmonic wavelength tuning, namely by adjusting the NPs, size, aspect ratio, corner-sharpness, heights, and even interparticle spacing [95,96,97,98]. Moreover, the progress towards more advanced LSPR sensors has led several authors to pursue the increase of polarizability of each nanostructure, typically through elongation and sharpening of NPs. Figure 2 presents some of the most common NP nanostructures. It is relevant to note that, as the NP anisotropy increases, the LSPR resonances are no longer purely dipolar, characterized spectrally by a single resonance band, but become affected by multipolar resonances, causing several resonant bands.

The interparticle coupling, represented in the lower section, affects nanoarrays and nanostructures, where the NPs are in proximity (or in contact), changing the ensemble spectral features, due to plasmon coupling mechanisms. More precisely, the plasmonic coupling can dramatically increase the field enhancement factors as interparticle spacing gets smaller [47,99,100,101,102].

If NPs are dispersed, their geometry is the main responsible to the way each field enhancement is created. Nanorods, being ellipsoids, present a great example on how the variation of a geometrical parameter influences their spectral features, e.g., the increase of a NR aspect ratio, and consequent anisotropy, causes the dipolar mode to split into two modes (a transversal and a longitudinal mode) with different refractometric sensitivities, absorption intensities and wavelength positions [103]. If more complex nanostructures are employed, even multipolar resonances can appear, as in the case of nanobars, or nanoshells [104,105]. In the past few years, a trend towards several polyhedral, disks, nanocrescent, and other complex nanostructures have been observed in the pursuit of higher RI sensitivities, and corresponding FOM [71,103,106,107,108,109,110]. The enhanced characteristics can be explained by the most elongated nanostructures, or the ones presenting sharper tips, generating hot spots. Moreover, these are spectrally associated with multipolar resonances with different refractometric sensitivities, as in the case of nanocrescents [103].

**Figure 3:**
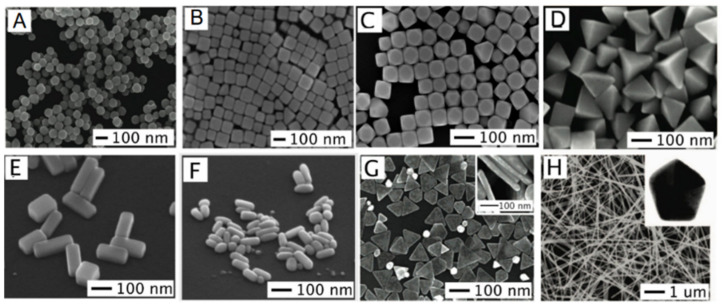
Typical nanoparticle geometries: (**A**) Spheres, (**B**) cubes, (**C**) truncated cubes, (**D**) right nanobipyramids, (**E**) bars, (**F**) spheroids, (**G**) triangular plates, (**H**) and wires. Reproduced with permission from [112].

Another important mechanism is related to the collective interactions between NPs, altering the optical response of the overall NP assembly. This coupling allows the creation of another plasmonic tuning mechanism, namely, by adjusting their interparticle distances, or period in arrayed nanostructures. This is of paramount importance in the spectral behavior of NP array and self-assembled nanostructures [111]. The creation of nanotriangles, nanorings, nanocrescents, and nanohole arrays using NSL, are such as case. Since fabrication starts with NS in suspension that later will self-assemble into HCP nanostructures. Thus, adjusting the NS size will produce different optical characteristics for each nanostructure. Moreover, among these nanostructures, the way the interparticle coupling occurs is strongly dependent on the anisotropy of the NPs, thus nanostructures as nanotriangle arrays will present higher RI sensitivities, than lesser anisotropic ones, as will be seen later. Some typical NPs often found in the literature are presented in Figure 3.

The shape-factor (ratio of the surface area of a non-spherical NP to a perfectly spherical one) of a nanostructure plays a critical role in the increase of dipolar polarizability, increasing as the NPs are made more needle-like [103]. Therefore, an NR of higher aspect ratio is expected to achieve refractometric sensitivity improvements [113]. Such higher refractometric sensitivities at higher wavelengths also apply for the generality of NPs and constitutes one of the great incentives to devise NPs capable of NIR operation for LSPR sensing. Then, for a specific nanostructure, higher RI sensitivities will be observed at longer wavelengths. On the other hand, for NPs of the same nanostructure, but composed of different nanomaterials, they will have different refractometric sensitivities, caused by their unique permittivity’s. This point will be specifically addressed in the next section.

**Figure 4:**
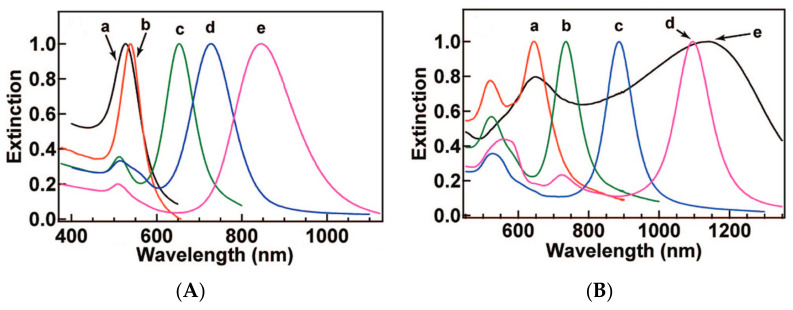
Wavelength dependency of the LSPR band on NP shape and size (**A**) Spectra a: nanospheres, b: nanocubes, and c–e: nanorods with aspect ratios from 2.4 to 4.6. (**B**) Spectra a–d: nanobipyramids with aspect ratios from 1.5 to 4.7, e: nanobranches. Reproduced with permission from [114].

It is possible to find in recent works the refractometric sensitivity dependency on geometrical parameters as shape-factor or aspect ratio [114]. Namely, NPs with a shape-factor of 2 (NS) exhibit the smallest RI sensitivity (44 nm/RIU), while nanobranches with the highest shape-factor exhibit the larger RI sensitivity (703 nm/RIU). Au nanobipyramids possess the largest FOM, which increases from 1.7 to 4.5 as the aspect ratio is increased from 1.5 to 4.7. In Figure 4 it is presented a redshift proportional to the increase of shape-factor.

Generally, the nanostructure and nature of the surrounding medium can have greater effects than those attributed to size on refractive sensitivities. In this section. geometrical and nanostructural factors and the way they affect the spectral response will be discussed in more detail for each of the NP geometries.

### 2.2. Spheres

If a census on NP nanostructures were to be performed, nanospheres would appear as the biggest representative, being at the same time the oldest geometry reported in the literature regarding NP-based plasmonics [115]. Generally, the NS size affects the spatial distribution of the polarization charges over the surface as well as the separation of positive and negative charges. Thus, this effect influences the peak maxima, resonance wavelength, and bandwidth, as shown elsewhere both theoretically and experimentally [116]. Small NS, ranging from 5 to 50 nm, are spectrally characterized by a solo absorption band. However, for larger NS (greater than 100 nm), multipole resonances can exist, caused by both parallel and anti-parallel movements of the charge regarding the incident EM field. Other LSPR modes can arise for non-spherical, NP are considered, as will be discussed with the other geometries.

**Figure 5:**
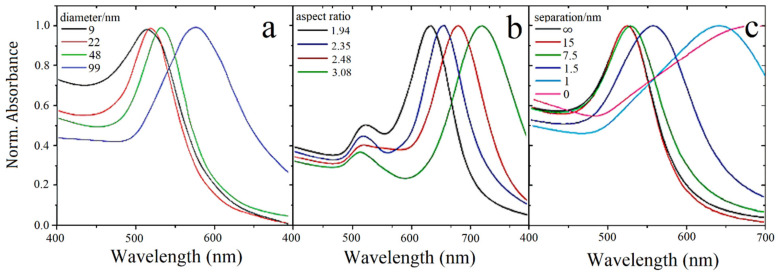
(**a**) NS diameter variation, (**b**) Ellipsoids of different aspect ratios, (**c**) NP volume fraction dependency. Adapted with permission from [117].

Nanospheres have intrinsic symmetries regarding the incident EM field orientation, causing only one mode that could potentially be easier to interrogate, while for non-spherical particles, such as NR, the resonance wavelength depends on the orientation of the electric field relative to the particle, and thus, oscillations either along (longitudinal) or across (transverse) the rod are possible, causing the resonances with different characteristics at two different wavelengths. In Figure 5 is reproduced the work presented in [117], where the particle size, deviation from spherical shape, and interparticle interactions have been compared for Au NP.

In Figure 5, it is possible to realize a direct relation between plasmonic resonance wavelength and diameter around 4.7 nm/nm. For ellipsoids, an absolute aspect ratio variation of 1 unit, corresponding to a considerably smaller variation, causes a 92 nm shift of the longitudinal mode towards higher wavelengths. Moreover, interparticle interactions also have a pronounced effect for volume fractions greater than 10%, resulting in a redshift and the expected broadening caused by dipole–dipole interactions.

To date it, in the literature, an immense use of Au NS and their property dependence upon geometrical and volume effects on the resonance band can be found, and since such geometries are now widely available, new compositions are being used, such as bimetallic NS, with a metal or dielectric core and metallic shell [105,118,119,120,121,122]. Although various bimetallic systems have been studied, with AuPd, CuNi and others, Au and Ag combinations are particularly common in the VIS-NIR range [123,124,125,126,127,128,129,130,131]. Regarding NIR regime, the authors of [118] recently published a work with Co-Ag and Co-Au bimetallic alloys studying shapes, sizes and composition dependency. They concluded that spherical-like shape performed poorly, in terms of sensitivity and Q-factors, when compared to cubic and rectangular nanostructures. Furthermore, the size is directly correlated to redshifts and widening of the FWHM values of the plasmonic band.

In 2017 the authors of [132] published a work with VO_2_ NS showing LSPR from 1200 to 1600 nm, filling the gap between the absorption ranges of Si and the LSPR of conventional TCO NPs (ZnO:Al, In_2_O_3_:Sn, etc.). In this study, the authors lowered the transition temperature of VO_2_ through Tungsten (W) doping. The presented results resolve the difficulties of using VO_2_ for practical NIR utilization, even though their performance in terms of Q-factors is around unity values.

The available works in the literature do not favor the use of this NP structure outside UV-VIS wavelengths because they perform poorly, and it is hard to achieve NIR wavelengths with them. Furthermore, if one tries to achieve this with these nanostructures, it requires the use of materials other than the traditional ones, or at least Ag/Au combinations, thereby potentially increasing synthesis difficulties, cost, and making them generally harder to use outside laboratorial scenarios due to, e.g., water reactions that could degrade their performance.

### 2.3. Triangles

Nanotriangles are NPs characterized by their sharp tips, presenting interesting plasmonic properties. Their lateral dimensions play a major role in the plasmonic resonance wavelength, being the major tuning parameter employed to shift the band from VIS wavelengths towards the NIR.

Until 2006, it was not possible to find in the literature resonances at wavelengths longer than 1000 nm using triangular NPs. This absence was overcome by Bastys et al. [133] with a report on the formation of Ag nanotriangles, synthesized by a modified photo-induced method, that operate at NIR wavelengths, up to 1491 nm. Later, in 2015, Wu et al. [64] published an alternative method capable of plasmonic operation at NIR wavelengths, by controlling the side-length of the Ag nanotriangles. Unfortunately, in terms of the maximum peak wavelength, this work could not achieve the near 1500 nm mark, caping at a maximum of 1234 nm, corresponding to the nanotriangles with 260 nm side length. The direct relationship between the side length and LSPR wavelength was found to be of $\lambda =3.1\;L_{side}\;+\;473$. So, to place the plasmonic band at 1550 nm, the nanotriangle must present a side length of 347 nm, which is hard to achieve with the seed-growth method used, since it required extremely low quantities of seed solution. Later, Kuttner et al. [134] synthesized Au nanotriangles through the seed-growth methodology where an overgrowth of Au could create these nanostructures with a 175 nm side-length, therefore presenting a significant redshift of the plasmonic band towards the NIR. The overgrowth methodology was further developed by Koetz et al. [135] by modifying the Au nanotriangles surface with smaller Ag NPs, creating the ability to tune the LSPR band from 800 to 1300 nm.

Overcoming the difficulties imposed by the colloidal methods can be achieved with NSL fabrication, as presented by Rahaman et al. [136]. The authors presented an interesting study showing how this fabrication technique can be used to produce LSPR sensors with Au, Ag, Cu, or Al nanotriangles from VIS to NIR wavelengths, by choosing a proper annealing temperature. Even biosensing applications can be found in the literature, such as the work presented by Soares et al. [137], with Au nanotriangles, showing RI sensitivities of 468 nm/RIU at wavelengths of 750 nm.

Recently, Chen et al. [138] produced a LSPR sensor at the NIR using copper deficient Cu_2−*x*_S nanocrystals (NCs) in triangular nanoplates geometry. The achieved FOM of the Cu_1_._81_S triangular nanoplates was comparable to Au NS. Moreover, the LSPR band presented stability regarding thiol reduction, a crucial characteristic for sensing at reductive environments. Finally, the authors tested the refractometric properties of the developed nanostructure to find an RI sensitivity of 716 nm/RIU at wavelengths between 1550 to 1750 nm. This resulted in one of the greatest sensitivities among the nanostructures presented to date at such wavelengths.

Another relevant study related to nanotriangles was presented by Wang et al. [139] with a numerical work on bow-tie nanostructures in a MIM configuration. This configuration presented significant enhancement factors, caused by strongly confined E-fields on a very small gap region. This configuration allowed wavelength tunability through UV to NIR wavelengths by changing the bow-tie inner angle, gap, period, and spacer distance. Moreover, these simulations yelled Q-factors around 3 for an operation between 900 to 1700 nm, along high RI sensitivities of 450 nm/RIU. Still, implementation difficulties will constrain the wide use of these kind of geometries.

There is even a subset of plasmonic NP called nano-antennas, and they are a hot and rapidly expanding research field. Existing works covering their structure and optical responses, such as [140], where the authors review the current state-of-the-art in fabrication and characterization techniques available for several types of nano-antennas. Bow-tie nanostructures are exemplary to show these nano-antennas properties. Their characteristic symmetrical sharp tips contrasts with NRs, which are usually referred to as a dipole nano-antenna. Studies with these nanostructures show great RI sensitivities. One such reported of these bow-tie nano-antennas is presented by Khoshdel et al., [141] to show sensitivities up to 923 nm/RIU and FOM of 5.5 at wavelengths up to 1620 nm.

### 2.4. Stars

This structure is a good candidate due to its plasmonic tunability and branching hot spots formation. Even-though not thoroughly seen in many works, these nanostructures can be found in the literature composed by Au and Au/Ag and synthesized with chemical/electrochemical approaches, nanoscale lithography, and EBL [142,143,144,145]. Now, works available in the literature do not show the existence of such nanostructures at Telecom wavelengths. The work that came more closer was the one present in [146] where was used Au nanostars presenting with two distinct LSPR resonances at around 700 and 1100 nm.

In 2014 Liu et al. [147] produced LSPR with Au nanostars that could be tuned from 557 to 760 nm. The tunability was achieved by adding 1 to 6 mL of 0.1 M HEPES solution, at 30 °C, with a redshift of 20 nm/mL. Nevertheless, with a FWHM of 450 nm for the 6 mL solution results in a mere Q-factor of 0.6. Advances were made in 2019 with the work presented in [148] with Au/Ag nanostars on an optical fiber tip. Even though this work only achieved a maximum resonance peak at 820 nm, it can be considered a step towards implementation of these nanostructures towards NIR sensing.

In 2020 Charci et al. [149] studied individual Au nanostars, separated from a flat gold film by a thin dielectric spacer layer, exhibit a strong light confinement between the sub 10 nm volume of the nanostars tips and the film. The studied found strongly polarized single, double, or multiple resonance peaks. The addition of the thin gold film was found to increase the scattering intensity, agreeing with the works done in slits configurations [150].

Works such as that presented by Barbosa et al. [151] on these nanostructures show that the sensitivities toward changes in the local RI increase for larger nanostars at the cost of lower FOM. In the same work, RI sensitivities between 215 and 316 nm/RIU were shown. As such, the performance of the smallest nanostars presented are comparable to those reported for nanorods (224 nm/RIU) and nanobipyramids (212 nm/RIU) with similar LSPR wavelengths.

### 2.5. Cubes

Nanocubes present two plasmon resonances, contrasting with the single resonance found for NS, due to the largely induced polarizations on both top and bottom facets. Spectrally, the resonance at a shorter wavelength, caused by the top facet interface, presents larger RI sensitivity than its counterpart [90]. This higher RI sensitivity in the top facet along with a lower sensitivity at the substrate interface favors its applications in both bio and chemical sensing. Alsawafta et al. [152] have shown a linear correspondence between plasmonic resonance and nanocube width of 0.00035 nm/nm (wavelength/side length). Therefore, larger nanocubes will have plasmonic resonance at higher wavelengths. Unfortunately, and as happens in the NS case, it would require such dimensions that will no longer allow the creation of plasmonic resonances, because the nanocube will have dimensions larger than one tenth of the incident light wavelength. Moreover, for the same volume, at VIS wavelengths, these nanocubes are located at longer wavelengths when compared to NS [153,154].

Ringe et al. [92] studied the LSPR frequency dependencies on size, composition, and substrate dielectric constant for the two most common materials, namely, Au and Ag. The study revealed a greater RI sensitivity for Ag by a factor of two when compared to Au. Bhatia et al. [118] recently studied rectangular CoAg and CoAu NP with LSPR peak wavelengths between 211 and 964 nm, and it was found that this geometry enhanced the absorption efficiency, leading to higher sensitivities, although the cube shaped NP produced an overall greater FOM. In terms of composition, the CoAg achieved better sensitivities when compared to CoAu bimetallic compositions.

Copper nanocubes were recently presented by Zheng et al. [155]. Even though Cu NP exhibits intense and sharp LSPR in the VIS region, the LSPR peaks become weak and broad when exposed to air or water due to the oxidation of Cu. SiO_2_ encapsulation could be a solution as performed in [156], yielding an aqueous Cu-SiO_2_ core-shell suspension with stable and well-preserved LSPR properties of the Cu cores. The cubic NP synthesized in this work exhibited a narrow and intense LSPR peak at 590 nm while the NR possess an LSPR peak at 700 nm and aspect ratio of 2.5:1. Unlike NS, the LSPR of the nanocubes and the longitudinal mode of NR are narrow and intense due to the shape effect. Therefore, creating a redshift of the LSPR from the inter-band transitions of Cu. Nevertheless, these works only provided solutions for the use of these cubic shaped NP in the VIS up to 900 nm, and not being able to achieve LSPR at Telecom wavelengths. So, it is unfortunately not fitting within the scope of this review.

### 2.6. Nanorods

Nanorods are very versatile nanostructures, both in terms of fabrication, LSPR band tuning, and spectral characteristics, finding several uses in the fields of chemical and biological sensing [157]. The most widely used nanomaterial for these NRs is gold, with seed-growth mediated methods as the most common fabrication procedure, enabling the easy tunability with precise control the aspect ratios.

**Figure 6:**
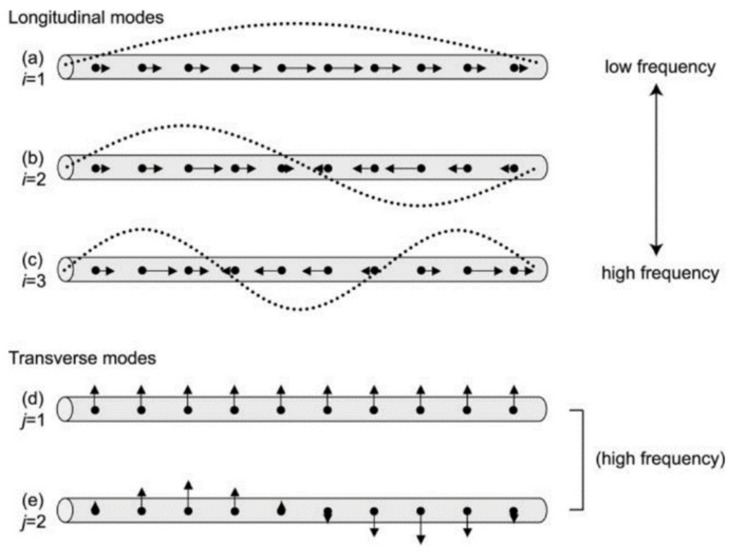
Fundamental and high-order LSPR longitudinal and transversal modes on NRs. Reproduced with permission from [158].

Spectrally, they show two resonant modes, namely a transversal mode, associated with oscillations along their width, and a longitudinal mode, caused by oscillations along their length. This is represented in Figure 6. Additionally, it is also represented by the higher order ones for these two modes which can also exist, although having less pronounced intensities.

Guo et al. [19] produced a very detailed review on Au NR at the VIS. That work extensively covered the theory behind their mechanisms, synthesis methods, optical platforms, and chemical and biological targets, so we invite the reader towards the article if a more profound knowledge about Au NRs is needed.

**Figure 7:**
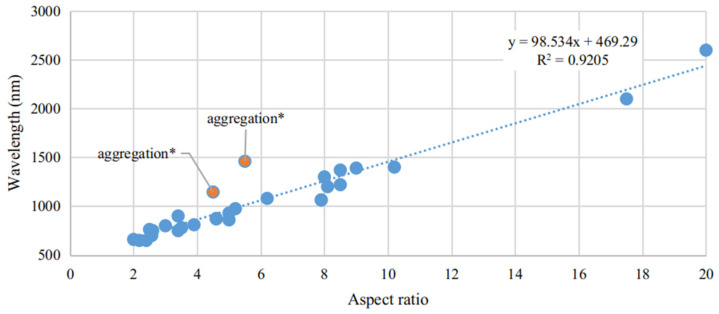
Longitudinal LSPR mode wavelength dependency on aspect ratio for Au NRs. Two data points are highlighted at a different color due to the displacement caused by aggregation effects.

As we move towards higher wavelengths, namely aiming for sensing around 1550 nm, the longitudinal mode is the one of interest, namely due to its higher wavelength position, increased refractometric sensitivity and resonance strength, when compared to its transversal mode. Moreover, since the length of the NR is the main parameter influencing its spectral characteristics, aspect ratio control is key to achieve this resonance at higher wavelengths, and as such we present in Figure 7 a compilation of some articles found with Au NRs, presenting the wavelength dependence on aspect ratio.

A linear relationship can be found between aspect ratio and resonant wavelength. The proportional factor found is just a little lower than what is presented in other works, where this factor is estimated to be between 95 and 115 [60,61,62,159,160]. The mismatch can be understood by the surface chemicals present, as in some works, still present significant CTAB concentrations at their surface, or by aggregation factors, which can shift the band, although typically to higher wavelengths, it presents a poorly defined behavior. This is highlighted in the figure, where due to aggregation of the Au NRs, the 1500 nm mark is achieved with lowered aspect ratios. Luo et al. [159] expressed the aggregation problem of NPs where it was shown that this effect produced a 280 nm offset, in the LSPR band, than what was predicted for Au NRs with aspect ratios of 20. The authors predicted a longitudinal LSPR mode at 2320 nm (by: λ = 95AR + 420 nm; AR: aspect ratio), whereas it was measured to be located around 2600 nm.

It can also be observed from the figure that aspect ratios between 10 and 11 would suffice to get the LSPR band at the wavelength region of interest. This can be done via the seed-mediated growth method, as presented in by Smitha et al. [37], reporting such synthesis of Au NR with a well-controlled aspect ratio between 2.4 and 16. Luo et al. [159], with Au NRs of aspect ratios up to 20, yielded a strong absorption band at 2600 nm. Wang et al. [63], with incredibly high aspect ratios up to 85.5, could be used to place the longitudinal LSPR mode from 1500 nm to 4450 nm. Finally, a mention to the work done by Tian et al. [161] must be made, due to the authors description on the synthesis of mono-disperse and high aspect ratios of Ag NRs, reporting lengths from 65 nm to 5 μm, corresponding to astonishing aspect ratio from 2 to 156.

The width of the resonance is also affected by the plasmonic wavelength, and was found be directly proportional to the wavelength, therefore causing a decrease in the Q-factor at higher wavelengths. This proportion was measured to be roughly around -0.007/nm. This can be seen from works as Gu et al. [54], who presented Au NR with resonances at 900 nm with a Q-factor of 5.5, to a Q-factor of 4.5 at 1000 nm, along works such as presented by Chen et al. [162] with Q-factors of 3.8 at 1050 nm.

Besides wavelength location of the LSPR band, the refractometric sensitivity of such nanostructures is of primal relevance. Works such as that of Chen et al. [163] present examples on how the optical and refractometric characteristics depend on aspect ratio by producing Au NRs with average aspect ratio of 5.2, exhibiting RI sensitivity of 366 nm/RIU. Truong et al. [164] showed a range of Au NR with aspect ratio from 2.2 to 3.9, with diameters between 12 to 16 nm, producing RI sensitivities between 201 and 298 nm/RIU. The sensor was then tested for the detection of the prostate specific antigen (PSA) biomarker, the lowest concentration recorded was around 1 aM ($6\times 10^{5}$ molecules), corresponding to a LSPR maximum wavelength shift of 4.2 nm.

Zhuang et al. [165] reported a work exploring large aspect ratio Au NRs (greater than 7.9), produced by seed-mediated method, exhibiting LSPR resonances at wavelengths greater than 1064 nm and RI sensitivities around 473 nm/RIU. However, the authors numerical work predicted RI sensitivities around 670 nm/RIU. This enormous difference was attributed to the surfactant capping layer on the NRs. Recently, Bala et al. [166] presented low aspect ratio Au NRs with RI sensitivities between 5000 and 6000 nm/RIU. These RI sensitivities are completely out of scale when compared to other studies, which can be explained by the nature of the surrounding medium, as done by Jain et al. [167]. In that work, it was predicted that the shielding of interparticle plasmon interaction by the dielectric medium, shown dependence on the surrounding RI. Therefore, these sensitivities cannot be directly compared to other works, which in most cases are composed of sets of calibrated standard RI oils.

**Figure 8:**
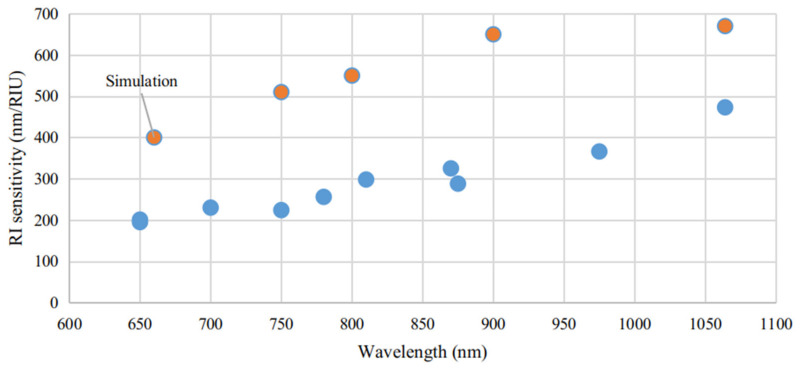
Longitudinal LSPR refractometric sensitivity dependence on aspect ratio for Au NRs. The orange data points are from numerical simulations, whereas the blue data points were experimentally measured.

RI sensitivity also presents a direct proportionality to the aspect ratio, and consequently to LSPR band wavelength, as can be seen in Figure 8.

The orange data points are prevenient from numerical simulations, whereas the blue data points were experimentally measured. While the numerical data presented an overall larger RI sensitivity than what was reported experimentally, their wavelength dependency is nearly of the same proportion, namely, around 0.66 nm/RIU/nm. This correlation between RI sensitivity and operational wavelength benefits the deployment of Au NRs for NIR sensing. To date, a wide variety of methodologies of synthesis and configurations enabled LSPR sensors development with Au NRs to present RI sensitivities and FOM, as 170–500 nm/RIU and 1.3–1.7, as reported in recent studies [16,163,168,169,170].

Not only Au NRs have been reported, and despite Au presenting the greater losses in both the VIS and NIR ranges when compared to Ag, it has the enormous benefit of being chemically stable in contact with water or air. Combinations of Au and Ag were produced in the pursuit of best spectral and physical properties of both metals. Briefly, when dealing with such nanostructures, the alloyed composition is used frequently to tune the LSPR band, e.g., when a higher Au percentage is employed, the LSPR band is redshifted [125]. An increase on Q-factors is also frequently observed fur such nanostructures when compared to single composition NPs, as shown by Wei et al. [171]. Chung et al. [125] also showed that these alloyed nanostructures also show increased refractometric sensitivities. Studies such as the one done by Dai et al. [172] with the development of bimetallic Au-Ag Core-Shell NRs superstructure tunable from 840 nm to 1277 nm, show the feasibility of these alloyed nanostructures to NIR applications.

**Figure 9:**
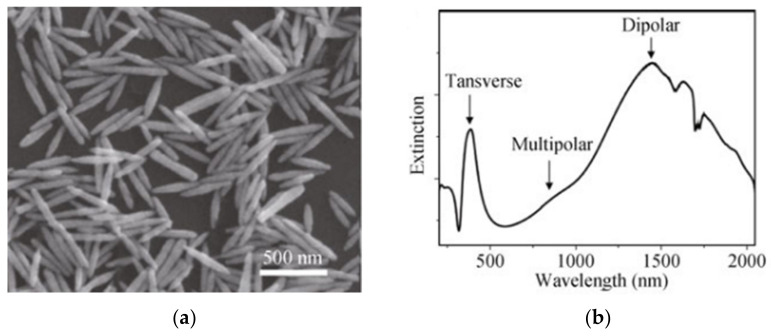
(**a**) SEM image of Ag nanorice; (**b**) spectral response of the nanorice ensemble showing longitudinal dipolar, multipolar, and transverse resonances. Adapted with permission from [174].

**Figure 10:**
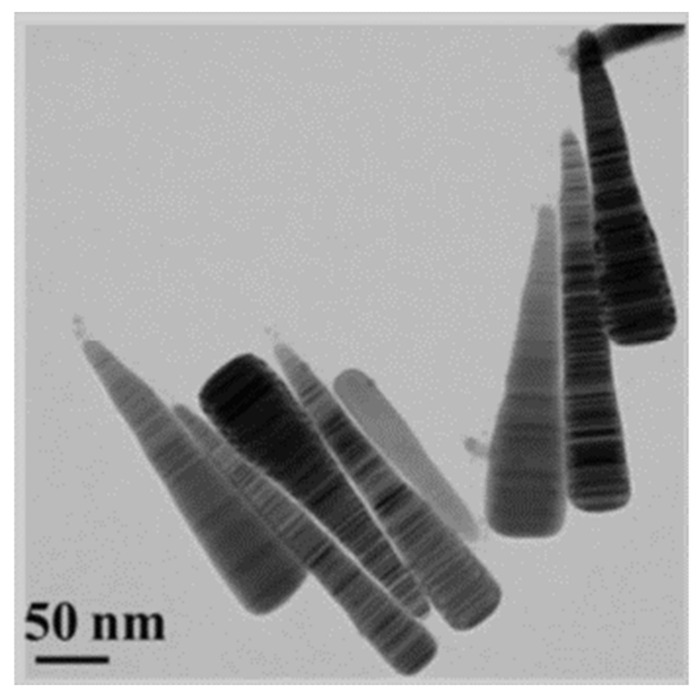
TEM image of silver nanocarrots. Adapted with permission from [175].

Recently, nanostructures similar to NR have appeared, such as nanorice and nanocarrots, which are represented in Figure 9 and Figure 10, respectively. Even though a lot less used than NRs they still present interesting spectral characteristics that could enhance some of the optical properties found on NRs [173]. Structurally, the nanorice structure differentiates itself from NRs mainly by it nearly smooth surface and round ends. Spectrally wise they present the same two longitudinal and transversal LSPR modes, with an additional multipolar resonance band. As happens with NRs, as their length increases, these longitudinal modes are shifted towards higher wavelengths [173]. Regarding the multipolar resonances, they advent from higher order longitudinal modes, as presented in Figure 6. These are less pronounced, as expected, and as can be seen in Figure 9b. Finally, the sharper tip of nanorice produces an additional shift of the resonance band, although the general plasmonic behavior is very similar to NRs.

In Figure 9b is presented a longitudinal mode around 1500 nm, matching well the purposes intended in this review. Nevertheless, the low Q-factors, i.e., the broader resonance is caused mainly from inhomogeneities in both size and shape distributions.

The nanorice refractometric characteristics are very similar to NRs, as can be seen from the work of Lopez-Tejeira et al. [176] who presented numerically a RI sensitivity of 547 nm/RIU at a wavelength of 800 nm, although with FOM greater than 20. Still, since in the literature only scarce reports of such nanostructures can be found, being mostly numerical, their synthesis methods to date have not matured significantly for us to state these nanostructures as viable options to perform chemical or biosensing at telecom wavelengths.

Regarding the nanocarrot nanostructure, as happens with NRs, its longitudinal mode wavelength depends on the aspect ratio, for which it can be tuned accordingly. This parameter is responsible for a linear shift towards higher wavelengths upon an increase in the nanostructure length. This effect is more pronounced for lower-order resonances as shown by Liang et al. [173]. A high RI sensitivity around 890 nm/RIU can be achieved numerically with these nanostructures. This is caused mainly by the sharp tip, as can be seen in Figure 10. Such high sensitivities make them very attractive for sensing applications, but as happens with nanorice, they still need better spectral and refractometric characterization, along with robust synthesis protocols capable of precise control over their geometry.

### 2.7. Rings and Trimmers

Other shapes exist with more exotic geometries, they can be produced by a variety of sequential methods, such as EBL, laser holographic lithography technology, colloidal or NSL, and NIL have been explored [23,75,177,178,179].

The non-colloidal methods offer great control over the nanoring array dimensions for which the LSPR band can be adjusted (mainly by variation of thickness and cavity sizes). An exceptional alternative was given by [180], where it was produced highly uniform colloidal Au nanorings and sphere-in-rings using wet-chemistry. To do so, the authors used circular Au nanodisks with different thickness and diameters as templates and assembling colloidal Au NS to them, they could produce Au nanorings.

Larsson et al. [23] produced a very interesting work both experimentally and numerically, regarding the optical responses from 75 to 150 nm diameter Au nanorings and compared their RI sensitivity response to Au nanodisks. The 75 nm diameter nanoring created a LSPR at 930 nm with a FWHM of 170 nm resulting in a Q-factor of 5.2, whilst the diameter of 150 nm produced a LSPR band at 1350 nm with a FWHM of 400 nm, resulting in a more degraded Q-factor of 3.4. The authors tested RI values from 1.33 to 1.42, for which was observed a sensitivity of 880 nm/RIU for the nanorings and 650 nm/RIU for the nanodisks.

Later, Hao et al. [181] studied the size dependency on the optical properties of thin Au nanorings and was shown a highly polarization dependent response. Toma et al. [75] presents another relevant work with Au nanorings, where the authors studied LSPR bands at NIR. The nanorings were fabricated by electro-deposition of gold nanorings onto lithographically patterned nano-hole array conductive surfaces. The authors produced LSPR bands that ranged from 1050 to 1650 nm with FWHM values of 219 nm for the shorter wavelength and 390 nm for the longer wavelength LSPR band, showing Q-factors of 4.8 and 4.2, and sensitivities of 805 and 1020 nm/RIU, respectively. The dimensions for the shorter wavelength LSPR nanorings have a diameter 146 nm, width of 124 nm with a separation of 750 nm. Regarding the largest nanodisk, it has a diameter 262 nm, width of 172 nm with a separation of 1000 nm. These nanorings stand along the highest reported sensitivities to date and, most importantly, they can be tuned into telecom wavelengths.

Regarding the optical response and geometry of trimmers, they are like nanorings. which makes sense to categorize them close to each other. A mentionable example of such trimmers is presented in [182] where the authors fabricated Au disk trimmers, using EBL, and studied their optical properties through VIS and NIR wavelengths. The structure showed double plasmon resonances. The main advantage of the trimmer structure is to provide a NIR LSPR with a high localized EM field. The dual-peak configuration possesses two different RI sensitivities of 130 and 375 nm/RIU for the shorter (around 650 nm) and larger wavelength (around 1350 nm), respectively. These sensitivities agree well with the works of other authors present in the literature for similar nanostructures [183].

### 2.8. Pillars

In 2010 Etin et al. [184] published their work with Au nanopillars system, merging SPR and LSPR effects to enhance RI sensitivity up to 675 nm/RIU with very sharp resonances of 6 nm FWHM. As a result, it offers the largest FOM of all nanostructures, standing with values around 112. In this work the assembly process was achieved using EBL as being able to produce both tall and high aspect ratio nanopillars.

Ten years later, in 2020, Zhong et al. [185] proposed a NIR multi-narrowband absorber numerically that could target Telecom wavelengths, by adjusting the period, thickness, radius and height of each pillar. Their work suggests Q-factors between 32 and 40, absorption coefficients between 83% and 87% at telecom wavelengths. To obtain such wavelengths with Au nano-pillars, the authors produced nanostructures with radius of 160 nm, periods of 350 nm, and heights of 1500 nm. These nanostructures could be produced using a relatively easy, even though costly, fabrication processes of one-step EBL first, followed by electrochemical deposition.

A near-identical to pillar nanostructures are nano-tubes, as presented by McPhillips et al. [186] with Au nano-tube arrays. This study showed these nanostructures to be capable of supporting LSPR. Moreover, that study also stated that an increase of the nano-tubes surface area augments the overall RI sensitivity, but with different magnitudes for the inside and outside walls interface. Nevertheless, the work only targeted wavelengths up to 800 nm, so still low to fit the purpose of this review work. Unfortunately, these nanostructures are not easy to find in the literature, and the authors could not find nanotubes at telecom wavelengths.

### 2.9. Shells

Nanoshells consist of a dielectric core with a coated metallic thin film, typically around 20 nm gold shell. These nanostructures can be tuned to higher wavelengths (up to 2000 nm) by increasing the core-shell ratio, as shown in several works [187,188]. With relatively small cores, the nanoshells have LSPR peaks in the VIS or NIR, and to obtain the band at Telecom wavelengths the core diameters must stand around 400 nm. The typical materials used for this kind of nanostructures are Au and SiO_2_.

Brinson et al. [189] were responsible for a step forward in the synthesis of both nanoshells and nanorice nanostructures, by reporting an easy method for Au layer metallization on the NP surfaces by using carbon monoxide as the reducing agent. This approach shown thinner and highly uniformity in regular shell layers, as well as high immunity to precursor or reagent preparation variations. Because of their work, it was possible to achieve plasmonic resonances at higher wavelengths. Nevertheless, this work only produced NP capable of reaching the 1200 nm wavelength mark. Later, Xie et al. [190] demonstrated an improved synthesis of hollow gold NS, providing a LSPR band tunable from 610 nm to 1320 nm. Even more recently, Preciado-Flores et al. [191] created a method which allows for the adjustment of both the inner diameter and wall thickness in a more precise way.

Unfortunately, to date there are not a substantial quantity of reports on nanoshells capable of supporting LSPR bands at Telecom wavelengths, and the sensitivities reported in the VIS are low when compared to the other geometries [192,193]. Two of the scarce examples on the use of nanoshells at Telecom wavelengths is the one reported in [3], where the authors reported an etched FBG functionalized with nanoshell NPs, as a refractometric sensor for RI values from 1.333 to 1.346. For that range, a sensitivity of 13.5 nm/RIU and optical power variation of 33 dB/RIU were observed. Unfortunately, this sensor obtains its RI sensitivity compromising the sensor robustness, due to the FBG etch to a mere 6 µm. The second example is the numerical work done in [55] with SiO_2_–Ag–Au and SiO_2_–Au–Ag nanoshells, where the work shows to have major complexities, from the fabrication precise control need of 1 to 2 nm thin outside metal layer and sensitivity of 270 nm/RIU with the band located at 900 nm for RI = 1.000. Therefore, for liquid mediums, the band cannot be located further than 1284 nm for a RI of 1.42.

### 2.10. Crescents

One of the more representative nanostructures which enhance its sensitivity by increasing the polarizability is the nanocrescent nanostructure [194]. These take advantages from Fano resonances originated from the interference between the quadrupolar mode supported by the horizontal crescent and the dipolar mode supported by the nanotip oscillating along the height direction [195]. The shape and dimensions of a nanocrescent need to be precisely designed to achieve localized field enhancement at the sharp edges, being the precise patterning of nanocrescent arrays the most challenging aspect because of the asymmetric geometry and the nanometer-scale tips. Electron beam lithography is usually the used method to construct these nanostructures.

Zheng et al. [195] shown that a nanocrescent gap separation highly impacts the optical response, being of primal relevance in the optimization of such nanostructures. With a gap distance smaller than 8 nm a Q-factor of at least 50 is obtained with a RI sensitivity highly linked to it, although with very minor values, around 22 nm/RIU. Nevertheless, Jiang et al. [196] shown greater RI sensitivities of 332 nm/RIU in a wavelength range of 1200 nm to 2000 nm.

The work done recently by Lin et al. [110] presented nanocrescents where both measurements and simulation of the transmission spectra for circular and linear polarization at normal incidence angle were made, reaching NIR wavelengths. At longer-wavelength resonances, of 1328 nm, the numerical results gave a FWHM around 205 nm, resulting in a good Q-factor of 6.5.

Other promising methods have appeared, aiming for increasing RI sensitivity. One of those examples uses plasmon hybridization [197]. This method consists of hybridization between localized and propagating plasmon modes in a coupled system, using NP arrays and continuous thin films, which will be discussed next. With the nanostructures that support hybridization lies nanocrescents and their doubles, nanocrosses and nanobars [153,198,199].

Finally, these nanostructures are mostly produced using electron beam lithography making all procedure to be potentially expensive and hard to tune, when compared to chemical methods. Nevertheless, it could pose as an interesting alternative to nanostructures for NIR in the future, even though it still needs development and characterization in terms of RI sensitivity, to produce more practical examples of LSPR sensors capable of mass production.

### 2.11. Arrays

Striding away from single and isolated NPs, when those are in considerably proximity, interparticle coupling occurs, changing the plasmonic resonance conditions. Thus, controlling this coupling mechanism can result in the design of extremely high local electric fields, also known as hot spots. Among such nanostructures, nanodisks arrays are of special relevance, since several works with nanodisks are present in the literature, ranging from on-chip to optical fiber solutions. Although only scarce reports have been found to reach telecom wavelengths, a mention to those works must be made [200,201,202,203,204,205,206,207]. Due to the nature of the available fabrication methods, usually via lithographic procedures, such as NSL, NIL, or laser interference lithography (LIL), just to name a few, these nanostructures are typically found in array configurations. It is also important to discuss the nanodisks spectral characteristics and their dependence on geometrical factors. These nanostructures belong to the group of nanostructures whose size, shape, and interparticle spacing impact strongly the resonance wavelength. The nanodisk diameter parameter is directly proportional to the LSPR wavelength on a ratio of 0.3 nm/nm (wavelength/diameter) as presented by Langhammer et al. [208]. Additionally, that study showed a LSPR band at 1773 nm for a 492 diameter nanodisk. The period between nanostructures in an array is also one of the major parameters to tune a LSPR band, showing a proportionality constant of 1.4 nm/nm (wavelength/Period), as presented by Lin et al. [201]. Moreover, the same study presented the wavelength dependency on nanodisk thickness to be inversely proportional with a constant of 3.2 nm/nm (wavelength/thickness).

Regarding RI sensitivities, the magnitudes obtained with these nanostructures surpass what can be obtained with nanocubes or NS. A great example is given by Lin et al. [201] where it is possible to asses a relationship between RI sensitivity and plasmonic wavelength. Namely, an increase of RI sensitivity with the increase of LSPR wavelength in a ratio of 0.292 nm/RIU/nm, presenting a sensitivity of 219 nm/RIU at 741 nm and a sensitivity of 345 nm/RIU at 1171 nm.

Reports such as the one presented by Rodriguez-Canto et al. [57] presented these nanostructures operating at telecom wavelengths with a 200 nm diameter nanodisks on an array with a period of 400 nm. Unfortunately this study presents mere RI sensitivities of 84 nm/RIU, nearly three times lower than the one presented by Lin et al. [201]. This difference is attributed to the functionalization used by the Rodriguez-Canto group and shows how functionalization can alter the refractometric sensitivities.

Aluminum nanodisks have also been presented by works, such as the one done by Langhammer et al. [208] with a production of Al nanodisks by a hole-mask colloidal lithography, with diameters ranging from 61 to 492 nm, resulting in LSPR bands capable of being tuned from 325 to 1650 nm. This work has the particularity of studying the effect of a passivation layer on these nanostructures. The authors found that, although a stable thin oxide of 2–3 nm (Al_2_O_3_) forms immediately after exposure to air on the nanodisk surface, this has less pronounced effect for the NP with larger volume nanodisks. Thus, an advantage for NIR sensing with this nanomaterial, when compared to VIS applications. The slightly lower volume density of this outer layer provokes swelling but has negligible impact in the overall particle dimensions and LSPR response. Therefore, as shown, these Al nanodisks can be regarded as a viable approach with a tunable behavior over the entire UV–VIS-NIR spectral range.

Recently, Chang et al. [71] showed great refractometric properties on a variation on the nanodisk array configuration by employing a MIM configuration. The produced Au-SiO_2_-Au nanodisk array was produced with nanodisk diameters of 1600 nm and a period of 2000 nm. These high valued parameters caused the LSPR band to be located at IR wavelengths (around 5000 nm), which is also responsible for the incredible high RI sensitivity of 1450 nm/RIU, which agrees well with the proportional constant of 0.292 nm/RIU/nm stated above, giving only a difference of a mere 20 nm/RIU. In the study was compared the effects of a single Au layer versus a double Au layer separated by a SiO_2_ layer and third tri-metal configuration, reporting RI sensitivities from 500 to 1300 nm/RIU corresponding to the single-layer and bi-layer configurations, respectively.

Finally, biosensing applications at NIR wavelengths have also been reported for such nanostructures, as in the work presented by Jiang et al. [209], at wavelengths up to 1025 nm measuring streptavidin, BSA, and Ig antibodies.

**Figure 11:**
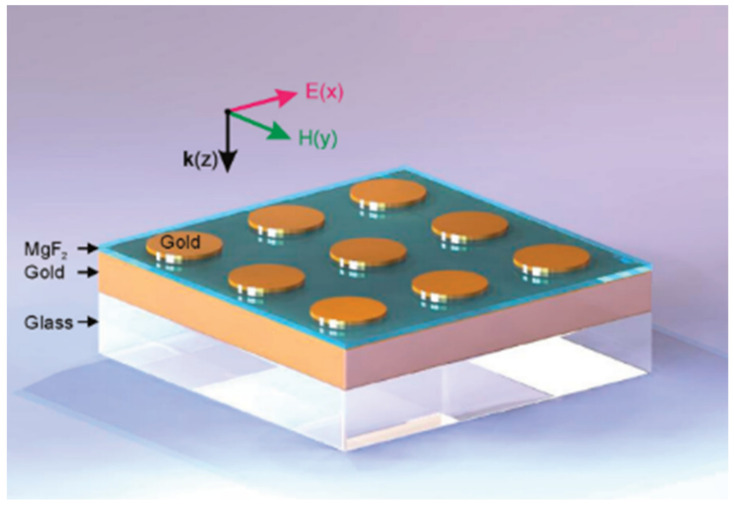
Nanodisk array over a 30nm MgF_2_ spacer, on top of an Au film of 200 nm. Reproduced with permission from [210].

Liu et al. [210] produced a high FOM LSPR device based on a nanodisk array configuration. Figure 11 illustrates the produced nanostructure.

In this configuration, the bottom layer role is to reflect the light to the top nanodisk array, enclosing the EM on the MgF_2_ layer, thus minimizing the nanostructure transmittance and enhancing the plasmonic resonances across the entire NIR. This design could be highly scalable and can be tuned from VIS to NIR regimes by changing the nanodisks diameter and period as before. The device was then tested as a refractometric sensor, achieving a RI sensitivity of 400 nm/RIU, standing around the same magnitude of what can be obtained using Au NRs. Additionally, the LSPR band located between 1615 nm and 1709 nm presented Q-factors around 5.3. Such a configuration, although interesting, since it combines LSPR phenomena with the field of perfect absorbers, does not enable easy interrogation using optical fibers, neither fabrication ease. Therefore, we cannot view such configurations to be in wide-spread use in biosensing using optical fiber technology.

Jensen et al. [211] also explored the array configuration with Ag nanotriangle arrays produced by NSL, allowing tunability from 400 nm to 6000 nm and narrow FWHM values. This was possible due to precise control of particle size, height, shape, and dielectric encapsulation of the nanotriangles using SiO_2_. Thus, causing an incredibly high tunability with redshifts of 4 nm/nm of film thickness. Even though NSL could impose challenges in the deposition on optical fibers, this fabrication topology could potentially enable a new series of highly tunable LSPR sensors at Telecom wavelengths.

Liang et al. [212] also produced an interesting work with an array of Au triangles embedded in VO_2_. Their work presented resonances between 956 nm to 1493 nm. The resonance peak showed a redshift with the increase of the VO_2_ films thickness, from 30 to 90 nm, producing a redshift of 810 nm. Thus, a proportionality factor of 13.5 nm/nm (wavelength/thickness).

Another relevant nanostructure for NIR plasmonic sensing is the nanohole array, usually fabricated by NSL, is among the easiest arrayed-like nanostructures to fabricate. These nanostructures have in general good uniformity, when compared with other geometries produced via purely chemical methods, along a general high sensitivity due to the combined effects of SPR and LSPR [213]. Therefore, nanoholes are likely to be seen in biosensing on field applications. Moreover as the plasmonic band is moved from VIS to NIR, the RI sensitivity increases as well [214].

Currently, there are some works exploring these nanostructures at the NIR and MIR, such as the work of Debbrecht et al. [215], who developed Al and ZnSe nanoarrays capable of placing the plasmonic band between 1.5 μm and 12 μm. The work also presented the typical tuning parameters to be of array period and thickness. Nanohole arrays have already been reported in the literature as the plasmonic element on an optical fiber tip, as presented by Zhao et al. [216] with a gold nanohole array LSPR sensor on a tip of an angle cleaved optical fiber, obtaining a FOM of 29 and a RI sensitivity of 487 nm/RIU at 800 nm. The authors presented an interesting method for nanostructure transfer to the fiber by a templated transfer method with a UV curing adhesive.

It is important to notice that, due to the lithography techniques involved, the RI sensitivity gain and LSPR wavelength tunability requires very precise and therefore costly mechanisms to produce such nanostructures. Thus, in general, this makes them not very suitable for wide use.

### 2.12. Superstructures

Not only size and shape of a specific nanostructure defines its spectral properties. usually their interparticle coupling, or lack of it, modifies the optical response of such nanostructure. Thus, superstructures may present interesting optical characteristics and a new method to achieve LSPR sensing at NIR wavelengths. These superstructures, often called as core/satellite superstructures, are just larger NPs onto which smaller NPs are coupled, that can be composed by the same nanomaterial and nanostructure, or not [217]. This allows to modify basic nanostructures optical properties by their surface effects.

The size or shape-oriented synthesis of such nanostructures is currently an intense area of study, as they can be fabricated either by template-assisted or template free self-assembly. The templated approach, typically involves inorganic templates to produce the desired nanostructures, while the later requires careful control over the capping ligands in colloidal interactions [218].

Another interesting aspect of the superstructure’s behavior, even though not yet at a mature stage, lies with the assembly and disassembly processes, which permit a reversible LSPR wavelength tuning. Regarding the plasmonic band location, we until this moment, unfortunately could not find examples of operation at the intended NIR wavelengths (near 1550 nm) [217,219]. A remarkable exception was presented by Dai et al. [172], with a bimetallic Au/Ag core-shell superstructured NRs, capable of tuning from 840 to 1277 nm, by varying the thickness of the Ag shell. Borwankar et al. [220] studied the spectral behavior on how highly dense protuberances on small Au NS changes its wavelength towards higher values, reaching wavelengths ranging from 700 to 1100 nm. Nevertheless, this configuration is not of special interest to biosensing applications, as to medical ones, due to the broad NIR plasmonic resonance and poorly reproducible behavior, which can be explained by the close particle spacing. Also the work presented very recently by Zhang et al. [221], who presented an AgTiS_2_ superstructure a capable of operation between 1200 and 2400 nm. The main tuning mechanism was the variation on the TiS_2_ thickness that redshifts the plasmonic resonance towards the NIR regime.

Moreover, since in this review we are concerned with obtaining nanostructures targeting high performance biosensing, a core characteristic is the RI sensitivity, to which we were not able to find meaningful material in the literature. Finally transfer mechanisms to optical fibers are also a requirement, and since these cannot be found in the literature, we are not going to explore more deeply the superstructures.

## 3. Materials

Considerable work in the nanomaterials field has been produced to shift the LSPR band from VIS to NIR [210,222]. Metals, such as Au, Ag, Cu, Al, and their combinations, reflect light very efficiently at VIS wavelengths, mainly due to the availability of free conduction electrons [148]. These are not only responsible for the optical properties, but for other physical properties as well, such as, heat or electrical conductivity.

**Figure 12:**
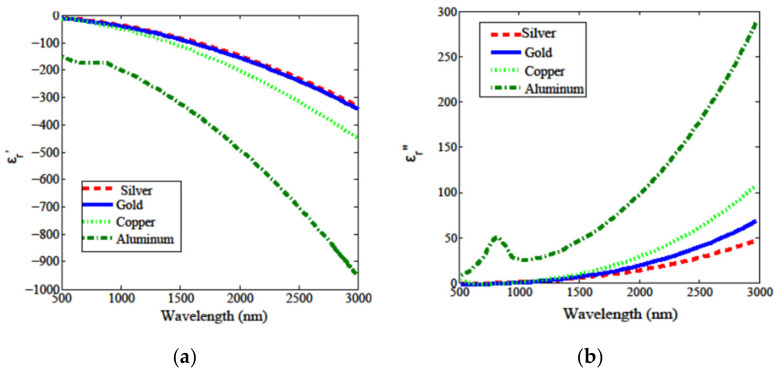
Permittivity of noble metals Au, Ag, Cu and Al: (**a**) real component; (**b**) imaginary component. Reproduced with permission from [223].

Figure 1 presents a very useful presentation of the increasing magnitude of the real permittivity for the metals when reaching NIR, common for all metals, and being the major drawback for the use of metals at the NIR for plasmonic based sensors. Figure 12 presents the complex permittivity of several common metal materials used in plasmonics up to wavelengths of 3 μm.

The metals useful for plasmonics must present a negative $\varepsilon^{\prime }$ in the range of interest, and for LSPR applications, this value must be within –20 to –1, along a small $\varepsilon^{\prime \prime }$, implying a large Q-factor (typically larger than 2, and preferably larger than 10).

Attending to these conditions, some metals use, become readily discouraged for plasmonics at NIR, such as Al, due to the high $\varepsilon^{\prime }$. Palladium and platinum possess also too much absorption, and lithium, that besides the fact that does not occur freely in nature, it is also highly reactive in the presence of water [224,225,226,227,228]. On the other hand, the unique Au resistance to corrosion properties makes it very appealing, constituting the main differentiation factor between Au and Cu. Additional practical issues, such as availability and ease of manipulation, especially for the fabrication of nanostructures, toxicity, durability, and cost also play an important role. Nevertheless, Au being the most resistant to corrosion, there will be combinations with Ag or completely other materials choices that are certainly the most promising in these categories and could potentially be the material of choice for applications beyond the 1300 nm range. However, Au and Ag, and their combinations, remain by far the most widely used metals in plasmonic applications, exhibiting plasmonic resonances in the visible and NIR ranges [117,224,229].

Other metals, such as Cd, Hg, In, Pb, Sn, and Ti, have their plasma frequency nearest to the UV wavelengths, so they do not present a good choice for NIR sensing [230,231,232,233]. Additionally, such metal particles are also readily oxidized, making experiments difficult, not to mention the existing high ohmic losses, that also severely reduce their performance. Therefore, plasmonic materials with lower losses than noble metals have long been sought, and in later years a lot more study is being dedicated to the pursuit of new materials supporting plasmonic resonances at NIR wavelengths [20,33,234,235,236]. The search for these elusive low loss alternatives, has spread to crystalline structured metals, inter-metallic composites, metal alloys, nitrides, and oxides. It is relevant to state that, even though the perfect material does not exist, and there is not an obvious material that can meet the ideal and required conditions listed above, their optical losses, occurring at different wavelengths could potentially dictate the use of a single material. In the following paragraphs it will be compared the low loss metals Au, Ag, Cu, and Al, and some other materials used in contemporary applications. Among these materials, Ag has the lowest losses in the VIS and NIR ranges and could potentially be a material of choice for these wavelengths [237,238].

### 3.1. Metals

There are an extensive number of relevant works using Au. Shalaev et al. [229] have shown experimentally a negative RI of –0.3 with an array of gold NR pairs, at telecom wavelengths, with a relative easy synthesis protocol. Showing the possibility of using Au in NIR plasmonics when using the right geometry, as shown in the previous section.

Works dedicated exclusively to the comparison between Al, Au, Ag, Cu, and Ni NPs can be found, such example is presented by Tan et al. [239]. The authors prepared Al, Au, Ag, Cu, and Ni NPs by laser ablation, comparing their size distributions impact on the utilized laser wavelength and energy, as well as, studying their optical characteristics. The resultant NPs presented two distinct peaks, one at the VIS (around 450 nm) and another one at NIR regions, namely at wavelengths greater than 1064 nm. Sharma et al. [187] compared Al, Au, Ag, and Cu in terms of sensitivity, SNR, and Q-factors for an SPR sensor. The authors concluded the Au to present the highest sensitivity, Cu the highest SNR and Q-factors, along better adhesion characteristics to optical fibers. Liu et al. [240] have shown a LSPR with Cu aiming for higher wavelengths, while providing solutions to the problem of hard synthesis. In this work was shown an easy and fast fabrication of colloidal Cu NP by laser ablation in liquid. Unfortunately, this research only provided resonance wavelengths up to 626 nm and did not solve oxidation issues.

Efforts to enhance the Cu properties in plasmonics have been achieved mainly by employing bimetallic schemes. Zakaria et al. [122] simulated and experimentally developed a bimetallic Ag-Cu thin film, and further deposited on a side-polished optical fiber. It was tested the RI sensitivity, showing a typical value of 108 nm/RIU for the bimetallic film, and for Cu only, a sensitivity of 425 nm/RIU. Furthermore, the oxidation problem was clear in the study as surface oxygen atoms increased the formation of alkoxides in the material. Research with bimetallic nanostructures had become increasingly relevant. Ke et al. [241] reported a method to produce mono-disperse and uniform Au/Ag NRs with a SiO_2_ protective shell, providing adequate protection against the environmental activities, showing re-usability and superior catalytic performances. These nanostructures were able to be tuned from UV to NIR by controlling their size or tuning the Ag shell thickness.

Recently, Bhatia et al. [118] presented, the effect of elongated bimetallic alloyed NP (Co-Ag and Co-Au) and their tunable plasmonic properties from deep UV to NIR wavelength ranges in terms of absorbance and sensing capabilities. Silver can support a strong plasmonic resonance from 300 nm to NIR wavelengths. Rycenga et al. [112] detailed vastly the Ag properties in plasmonics, namely the resonance wavelength tunability, with respect to size and geometry. Dependencies such as in a polyhedral shape, decreasing the corner sharpness, not only shifts the resonance peak towards higher wavelengths, also makes it more sharp as well, therefore increasing its Q-factor. Silver is the metal with the lowest losses in the VIS and NIR ranges [41].

Bauch et al. [214] investigated the optical properties by combination of a nano-structured 14 nm Ag film, embedded between thin films of TiO_2_ and AZO, resulting in a plasmonic band at NIR wavelengths with minimum transmittance of 1.5% at 1500 nm. The dipole excitation by the nanodisks, caused significant broadening (FWHM greater than 1000 nm) and a redshift due to the interaction with the whole array. These results do not provide a good enough basis to build plasmonic sensors for RI sensing due to the poor Q-factor obtained. It should be emphasized here that these sensors with Ag oxidize very easily, and they could only be used for a single analysis.

Nickel NP have been reported with plasmonic bands on the UV spectrum [242,243]. These Ni NPs find uses as potential adsorbent in sewage treatment process and removal of cationic and anionic triphenylmethane dyes, and reinforcement in polymer matrix composites [120,244,245]. Other works, such as that presented by He et al. [246], have combined Ni with TiO_2_, reaching plasmonic resonances at 750 nm, by changing the Silica NS size on the HCP ensemble, although the complexity in fabrication, more precisely due to the need of the awkwardly large Si NS, makes this approach cumbersome and difficult to apply them on optical fibers plasmonic sensing systems [247].

Among the alkali metals, Na and K present the lowest losses, even more than those of typical metals, as Ag [224]. In the last decade, Li, Na, and K started to find uses in solar cell applications, by their utilization as an additional donor material in organic solar cells [248,249,250,251]. These works suggest that doped CuO NP with these alkali metals show significant potential in optoelectronic applications, but unfortunately cannot be applied for optical sensing purposes [252,253]. A consequence from their highly reactive interactions with air and water. Therefore, not a good choice for optical sensors.

Wang et al. [236] also studied Na properties, by taking advantage of its low melting point, producing a stable Na film plasmonic device. This work produced a FOM at 1300 nm, four times greater than what is achievable with Ag for the same wavelengths. Additionally, the Na film was covered with a dielectric film, therefore protecting it from oxidation.

These alkali-metals can be considered theoretically high performance plasmonic materials at NIR wavelengths, due to their permittivity characteristics and less than half of intra-band losses when compared to Ag. Nevertheless, these alkali-metals have not affirmed themselves due to practical considerations upon fabrication and stability with the environment.

### 3.2. Transparent Conductive Oxides

Wide-bandgap semiconductors could perform at NIR as well as Ag and Au at VIS wavelengths, by their combination of highly compacted modes with low losses at Telecom wavelengths. Therefore, a possible solution to the loss problem at NIR could involve the use of transparent conductive oxide (TCO) materials as shown by Franzen et al. [254]. Heavily doped degenerate semiconductors such as tin-doped In_2_O_3_ (ITO), aluminum doped zinc oxide (AZO), indium-doped CdO (ICO), titanium dioxide TiO_2_ and gallium doped zinc oxide (GZO), being conductive and optically transparent at VIS wavelengths, support plasmonic resonances at Telecom, and longer, wavelengths [255,256,257,258]. Moreover, the free carriers concentration of these materials causes the plasmonic band to be located at higher wavelengths [20]. Additionally, these materials allow the carrier density to be adjusted and thereby the LSPR energies can be adjusted to cover NIR and MIR regions.

Conventional semiconductors, such as Si, Ge, Al, Ga and alloyed combinations, as well as phosphides, arsenides and antimonides may be grown as highly doped nanocrystals, and unlike metals, these can show small $\epsilon^{\prime }$ and very small losses at NIR and MIR. Moreover, according to the Drude model, heavily doped semiconductors present a near absence of inter-band transitions [259].

A mention must be made of Lie et al. [130], with their development of $Cu_{2-x}Se$ NS synthesized via a simple templated method exhibiting strong LSPR in the 980 to 1300 nm wavelength region. Back in 2010–2011 Naik et al. [259,260] discussed these materials, encouraging the use of heavily doped semiconductor plasmonics at telecom wavelengths. However, the authors observed that doping optimization is still a challenging theme due to the fact that higher doping can cause low donor ionization efficiency and solid solubility problems, as is stated in other review articles [33,261,262,263]. In 2010 as well, the authors of [20] compared the losses in AZO and GZO at telecom wavelengths, concluding that those are less than four times those of Ag.

**Figure 13:**
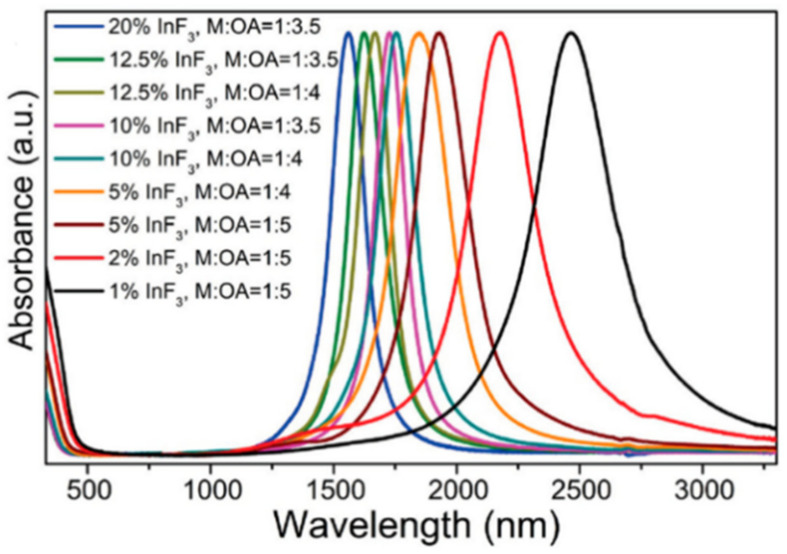
UV-VIS-NIR spectra of spherical FICO NCs dispersed in CCl_4_. Reproduced with permission from [264].

In 2014 Ye et al. [264] presented a generalized doping methodology for the synthesis of mono-disperse, doped metal-oxide NC exhibiting LSPR at NIR with the highest to date Q-factors. The work explored the use of a fluorine atom as a dopant material replacing oxygen atoms as a donor. The synthesized fluorine and indium co-doped CdO NCs and fluorine and tin co-doped CdO NCs displayed remarkably Q-factors and capable of tunability between 1500 and 3300 nm. Figure 13 shows the tuning of the LSPR band dependency on InF_3_ percentage.

The InF_3_ percentage variation from 1 to 22.5% caused variations in wavelength from 2456 to 1544 nm. Nevertheless, the tunability component in NC size becomes limited at higher InF_3_ concentrations (greater than 10%). Regarding the synthesis method, the mono-disperse NCs were synthesized by thermal decomposition of cadmium acetylacetonate and indium fluoride in a mixture of oleic acid and 1-octadecene.

More recently, in 2019, Liu et al. [265] explored the tuning of MIR plasmon resonances by introducing p-type dopants (Cu^+^ or Ag^+^) into n-type metal-oxide nanocrystals through cation-exchange reactions. That work shown the possibility of achieving resonances located between 2000 and 3000 nm. Yet, the authors acknowledge that the available dopant types and their concentration remain limited for many metal-oxides. This can be attributed to difficult kinetics reactions promoting dopant incorporation, thus not solving the issues reported in previous works, such as [264].

**Figure 14:**
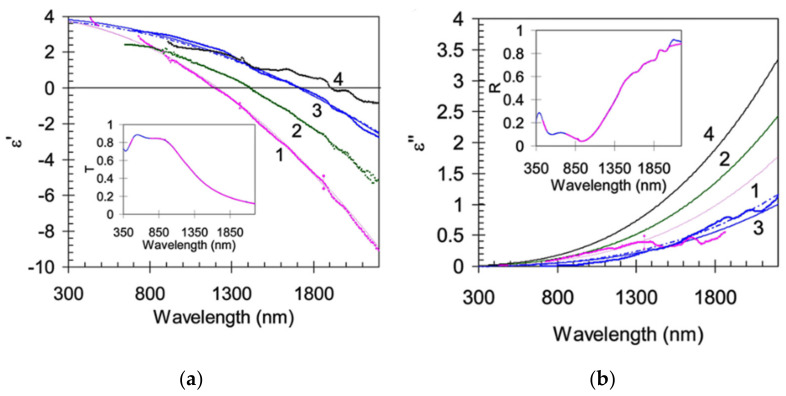
Experimental spectra of real (**a**) and imaginary (**b**) parts of electric permittivity. ITO (1), ZITO (2), AZO (3), and ITZO (4). Reproduced with permission from [266].

Regarding inter-metallic materials nitrides present lower losses along a higher carrier concentration, when compared to silicides and germanides. However, at NIR their inter-band transitions are not eliminated effectively, thus, making them less attractive as plasmonic materials [259]. In 2011 was produced a very interesting work with ITO, AZO, ZITO, and ITZO epitaxial films (thickness between 100 nm to 270 nm) grown using the pulsed laser deposition [266]. The obtained $\varepsilon^{\prime }$ and $\varepsilon^{\prime \prime }$ components for these four materials are presented in Figure 14.

**Figure 15:**
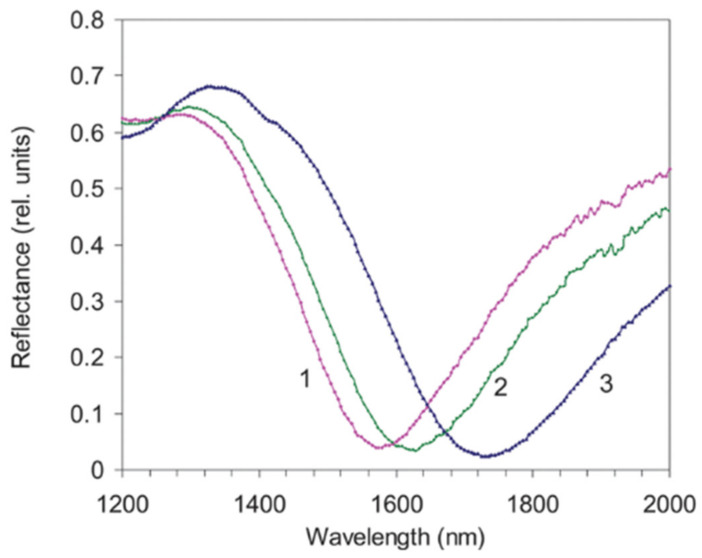
ITO reflectance deposited on a glass substrate (*n* = 1.510) for θ = 57.2°, 55.2° and 52.5°, corresponding respectively to the 1,2 and 3 curves. Reproduced with permission from [266].

The important result was the obtained zero value of $\varepsilon^{\prime }$ between 1190 nm and 1910 nm, therefore positioning the plasma frequency at the wavelengths of interest. The reflectance spectra produced from the ITO deposited onto a glass substrate (*n* = 1.510), and within the Kretschmann configuration, by varying the incident wavelength is reproduced in Figure 15.

The study showed metal-like characteristics with low losses in the 2000 nm region, even though with higher FWHM values (greater than 800 nm), and a very asymmetrical spectral shape. Moreover, the losses found were still lower than the ones found in typical metallic nanomaterials in the range from 1500 to 2500 nm.

**Figure 16:**
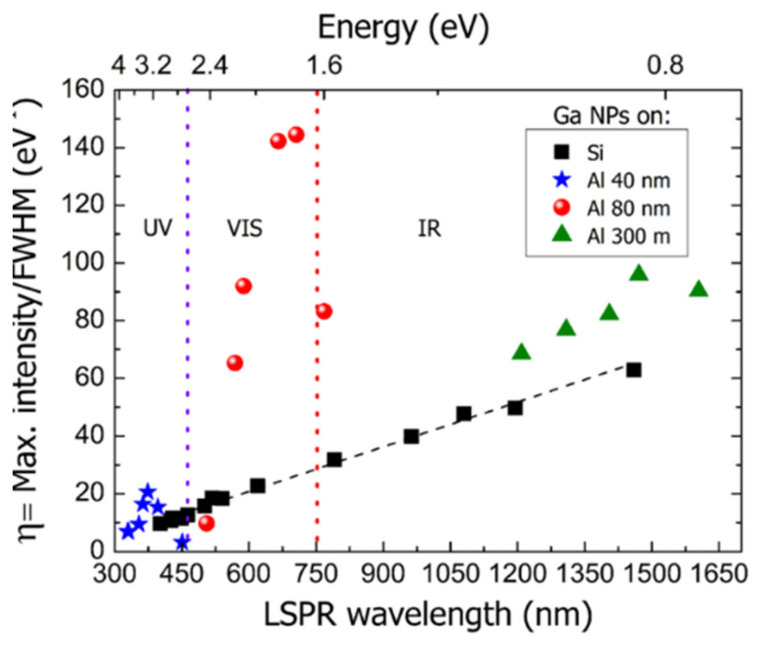
The figure of merit, η (calculated as the maximum intensity over the FWHM) show higher values for longer wavelengths, for Ga NPs is presented for Al and Si templates. Reproduced with permission from [273].

Gallium NP have been reported in literature with applications from battery management field to biosensing. They can be fabricated by various methods and conjugated with optical fiber technology [267,268,269,270,271,272]. Gallium NPs that were able to achieve LSPR between UV to NIR with Al nanostructured templates were presented in 2020 [273]. The patterned templates were prepared by anodization process on high-purity Al foils. The results are reproduced in Figure 16.

These NPs spectral characteristics shown enhancements for longer wavelengths, namely in the NIR region, presenting another factor favoring the use of such materials in plasmonic sensing applications at NIR. Furthermore, this work confirmed an outstanding improvement, over previous works, in terms of LSPR intensity, tunability and FWHM [274].

In 2019, Soumya et al. [275] took a step further in the fabrication cost of AZO, AIZO, and IZO TCOs films for plasmonic applications in NIR region produced by spin coating. Their properties were found to be consistent with those fabricated by sputtering, vapor–solid or pulsed laser deposition [266,267]. These were produced to have a specific carrier concentration around $10^{20}/$cm^3^, necessary for plasmonics in the NIR range.

Various works with ITO have been done recently with promising results in the NIR range and aiming for very distinct technologies, such as the study presented in [276] that compared different concentrations of ITO into PMMA, forming a composite with low transmissivity at wavelengths greater than 1000 nm. This study aimed for the use of this composite for IR Filtering on auto-mobile industry, namely, for vehicle windscreens. ITO enables tuning of the resonance wavelength through changing its carrier concentration.

**Figure 17:**
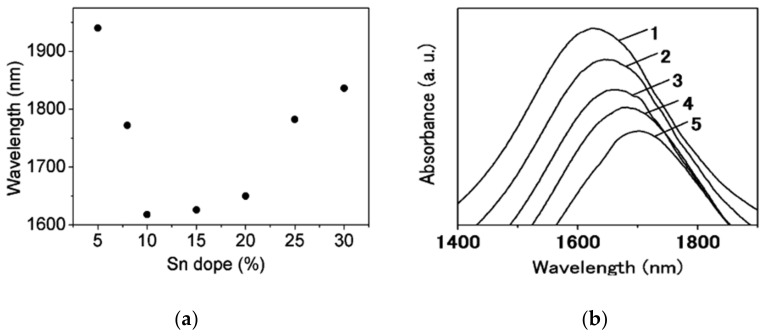
(**a**) Dependence of the plasmonic resonance of ITO/Sn alloyed NPs for different Sn concentrations. (**b**) RI sensitivity for the 8% Sn concentration NP. Curves 1 to 5 represent the following RI values: 1.37, 1.42, 1.48, 1.55 and 1.55. Reproduced with permission from [277].

Kanehara et al. [277] produced alloyed NP of Sn and ITO and characterized their spectral characteristics as the In/Sn molar ratio varied. Their plasmonic band varied from 1626 to 2200 nm, presenting a blue shift as the Sn concentration increased, up to 10%, and a redshift for Sn concentrations above 10%. The authors also tested its RI sensitivity for the alloy with 8% Sn to be of 422 nm/RIU, as presented in Figure 17.

The Sn percentage on the alloy impacts the plasmonic resonance wavelength, mainly by increasing the electron density of the nanomaterial, reaching a maximum at 10% concentration. Above this value, impurity and phonon scattering causes the charge mobility to decrease [278].

This work agrees well with others present in the literature, such as with [279], where colloidal ITO NC, stated that tunability can be achieved through control of Sn doping ratio, allowing this to be a good platform for RI sensing at NIR and MIR. The work resulted in LSPR band at 1750 nm with FWHM of 400 nm, therefore a Q-factor greater than 4. The authors of [280,281] presented a study on the behavior of LSPR with ITO NC when doped with Sn, in the 1400–5000 nm wavelength range. Similar works with LSPR metal oxide NC have also presented good results in the NIR to MIR range [282,283,284,285].

### 3.3. Transition-Metal Oxides

Transition-metal oxides (TMOs) are also interesting candidates. Tungsten trioxide (WO_3_), dioxide Vanadium (VO_2_) and rhenium trioxide (ReO_3_) are some among the available options for plasmonics [286,287,288,289,290,291,292,293]. The later material major drawback advents from its price, being among the most expensive metals, limiting practical applications. VO_2_, extensively studied due to its excellent metal–insulator transition properties, display a reversible phase transition at 68 °C [132,294], thus reflecting very well IR light at temperatures below 68 °C and transmitting well above that [295,296]. These thermal properties of Vanadium are explored in a new trend of smart materials. It is possible to synthesize Vanadium dioxide NP by several techniques, including chemical vapor deposition, sol–gel synthesis, sputter deposition, and pulsed laser deposition [297,298].

Rini et al. [287] studied VO_2_ NC at the desired optical Telecom window. The NP were grown by ion implantation and self-assembly in Si. Their size and shape were controlled by a variation of the annealing time. It was achieved an absorption band at 1550 nm with a 20% magnitude. Later in [291] it was developed a two dynamically tunable MIM wave-guides, employing (VO_2_) thin films as the active element, working on wavelengths of both 1310 nm and 1550 nm. The polyvalence properties of Vanadium also led to a mixing of this material with Au and Ag to shift the band gap to the VIS, as done in works like [212,289,299,300]. Moreover, despite the Vanadium interesting properties, and works with Vanadium NP in SiO_2_ it is still a need for more research into turning them a useful plasmonic material [301].

Manthiram et al. [286] studied WO_3_ as a good LSPR nanomaterial at VIS and NIR wavelengths. The authors shown that tunability is possible through heating of the NPs in an oxidizing environment. Finally, the produced NPs were tested as a refractometric senor, showing a RI sensitivity of 200 nm/RIU, at wavelengths around 1100 nm.

It is possible to conclude that, doping wide-bandgap semiconductors can tune them to perform well at NIR wavelengths, where they present lower losses than typical plasmonic metals, such as Ag or Au. This is mainly caused by the absence of bound-state transitions at their bandgaps.

## 4. Discussion

Recent nanomaterials and nanostructures developments produced enormous improvements in the field of nanoplasmonic sensing, enabling an increase of refractometric sensitivity along a better control over plasmonic wavelength tunability.

As reported, the coupling condition for a plasmonic resonance to occur is dependent on the medium and NP permittivity’s, along geometrical and interparticle coupling parameters, as was explicitly stated in Equations (1)–(4). Accordingly, we divided this review on the nanomaterial and nanostructural elements to study each parameter impact on the overall LSPR band spectral characteristics, while presenting a few of the more relevant studies reported recently that contribute to the development of the targeted optical C and L-band communications band.

The complex permittivity of each material is unique, presenting different absorption intensities on plasmonic resonances, at specific wavelengths. Focusing on the loss parameter, the nanomaterial choice must be done taking into consideration its $\varepsilon^{\prime \prime }$ parameter, which must be as small as possible at the wavelength of interest. For instance, at 1.5 μm, among the typical metals, aluminum is the one presenting highest loss with a complex permittivity $\varepsilon^{\prime \prime }=46$, followed by copper (14), gold (12) and silver (5). Thus, the latter in theory will present the highest Q-factors at this wavelength and its use should be promoted. Regarding the permittivity real component, as presented in Figure 13, aluminum is again by far the worst performant nanomaterial, and the same ordering repeats, with silver coming as the best potential material for the intended wavelength range. Nevertheless, the unique Au resistance to corrosion makes it very appealing for sensing purposes, since it presents the most stable and durable characteristics. Additionally, the availability of matured synthesis protocols, which enable the NPs to be fabricated with high controlled geometrical properties gives the advantage to gold, with respect to the other four metals. Nevertheless, silver was also found to be a very common material in the fabrication of NPs, although the small increment in performance in many cases does not justify its use, since it presents the drawbacks just enunciated. To mitigate oxidation, and in general physical degradation, works such as that of Ke et al. [241] present SiO_2_ protective shells solutions, with minimal wavelength shifts, which could present a viable response to address reproducibility and stability issues when performing sensing with plasmonic based NPs. In some cases, gold is even reported to present better refractometric sensitivities than silver, as presented by Sharma et al. [187]. Finally, Au and Ag combinations were found extensively, to pursue wavelength tuning and spectral characteristics enhancement at NIR wavelengths. These bimetallic alloys also allow for an extra tuning parameter, namely the Ag thickness decrement on the Au NP will redshift the band towards higher wavelengths [125].

Transparent conductive oxides, and particularly ITO, AZO, ICO GZO, and TiO_2_, as well as highly doped semiconductor nanomaterials, due to their high free carriers concentrations, were shown to support plasmonic resonances at Telecom, and longer, wavelengths [255,256,257,258]. Typically, their carrier concentration is a parameter that is often adjusted to place the LSPR band at a specific wavelength, a parameter that cannot be adjusted in pure metals. Moreover, the nanomaterials exhibit small $\varepsilon^{\prime }$, very small losses and an almost absence of inter-band transitions at NIR and MIR [259].

Figure represents the complex permittivity for some common TCOs where it is possible to see that all of them present better characteristics than what can be obtained by means of common metals. If one only consider their physical properties they present themselves as the more natural approach for LSPR at 1.5 μm. Currently works such as the one presented by Ye et al. [264] on doping methodologies for TCOs reported LSPR bands between 1500 and 3300 nm, which the highest Q-factor achieved to date. Kanehara et al. [277] with the Sn doped indium oxide NPs also shown LSPR between 1626 to 2200 nm, with Q-factors greater than 4, and a refractometric sensitivity of 422 nm/RIU. Thus, a highly performant nanomaterial at the wavelengths of interest.

Finally, TMO, namely WO_3_ and VO_2_, have also been reported as interesting and viable candidates for NIR sensing, although with poor stability when in contact to typical environmental conditions [286,291].

To present viable plasmonic nanostructures capable of having LSPR bands at Telecom wavelengths, are not sufficient to discuss only the nanomaterials physical properties, since we would declare TCOs and TMOs as the only viable highly performant nanomaterials, which is not completely true. Although they must be considered as promising nanomaterials, to date, they are not the most representative nanomaterial for the intended applications and are not expected to be soon, mainly due to their availability, cost, in some cases toxicity, but most of all due to their chemical stability to environmental conditions, which will impose a hard to overcome barrier.

Now, a look into the interesting field of nanostructures will be given, since through a stringent choice of NP geometry will present spectral unique spectral features, which could be even further enhanced through interparticle coupling, as happens for array nanostructures or superstructures.

Starting with NS, especially the ones made of gold, are found to be the most representative nanostructure to date, mainly caused by their simplest to fabricate profile. Their main tuning mechanism lies with its size and presents a single LSPR band. The use of metal NS would become cumbersome to work with at NIR wavelengths, since it would require enormous NPs. More precisely, and as represented in Figure 5, the wavelength depends on diameter on a proportion of 4.7 nm/nm. Thus, for operation at 1.5 μm, it would require a gold NS with a diameter of 8850 nm. As such, almost all works reported present this nanostructure to operate at VIS wavelengths and with the lowest RI sensitivities among nanostructures, presenting values around 44 nm/RIU. Nevertheless, the use of other nanomaterials as TCO or TMO allowed them to operate at NIR and even MIR, as in the notable case of the alloyed ITO/Sn nanomaterial. Kanehara et al. [277] presented such an example, with a plasmonic band operating from 1626 to 2200 nm, along an acceptably RI sensitivity of 422 nm/RIU. Finally, this nanostructure will always perform poorly when compared to the others in terms of RI sensitivity and Q-factors due to their inability of generating the so called hot-spots.

Nanotriangles on the other hand present sharp tips which enable them to generate hot spots at their tips and enhance refractometric and spectral properties in comparison to NS. Nanotriangles lateral dimensions is the major tuning parameter to move the LSPR band. The direct relationship between the side length of a Au nanotriangle and its LSPR wavelength was found to be of $\lambda =3.1\;L_{side}\;+\;473$, as reported by Wu et al. [64]. So, to place the plasmonic band at 1550 nm, the nanotriangle must present a side length of 347 nm. This could be somewhat achieved by an overgrowth methodology, as presented by Koetz et al. [135], more easily by NSL, as presented by Rahaman et al. [136], or another lithographic methodology. Regarding their refractometric properties, works such as the one presented by Soares et al. [137], with Au nanotriangles, have shown RI sensitivities of 468 nm/RIU at wavelengths of 750 nm. This surpasses by a factor of 10 what can be achieved with Au NS, or with the more exotic TCO NS at lower wavelengths. This suggests that, since RI sensitivity increase with LSPR wavelength, if a nanotriangle structure was to be devised for operation at 1500 nm, its RI sensitivity will surpass by far what was achieved with the ITO/Sn NS. Moreover, Chen et al. [138] achieved a RI sensitivity of 716 nm/RIU at wavelengths between 1550 to 1750 nm with Cu1.81S triangular nanoplates, although not the best in terms of stability, it shown good refractometric properties. Finally, nanoantennae should be mentioned as capable of highly sensitive refractometric sensing, as presented by Khoshdel et al. [141], with sensitivities up to 923 nm/RIU and FOM of 5.5 at wavelengths around 1620 nm. This work, although numerical is a great example of how interparticle plasmon coupling can enhance their sensing characteristics.

Nanostars even though presenting an interesting nanostructure and capable of generating the highly regarded hot spots, to the best of our knowledge, there have not been reports at the wavelengths of interest in this review. This does not mean that in the future there will be no reports proving the contrary. It only means that it is an area that could be more explored in the future, and perhaps present interesting LSPR characteristics at the NIR. Nevertheless, Barbosa et al. [151] presented Au nanostars operating around 850 nm presenting RI sensitivities between 215 and 316 nm/RIU, which are comparable to those reported for Au NRs (224 nm/RIU) and Au nanobipyramids (212 nm/RIU) at similar wavelengths. Thus, this nanostructure could effectively be a viable solution for sensing applications at NIR, with comparable sensitivities to other nanostructures composed of the same nanomaterial.

Nanocubes, spectrally wise present two LSPR bands. Namely, the resonance at a shorter wavelength (caused by the top facet) presents larger RI sensitivity than its counterpart [90]. This higher RI sensitivity in the top facet could present interesting properties for sensing applications. Due to their symmetries, the only geometrical parameter to tune its plasmonic response is the side length, presenting a proportion of 0.00035 nm/nm (wavelength/side length), as presented by Alsawafta et al. [152]. Thus, for operation at 1500 nm, and as happens in the Au NS case, it would require such dimensions that will no longer allow the creation of plasmonic resonances, because the nanocube will have dimensions larger than one tenth of the incident light wavelength. As so, gold nanocubes will not be able to work as a viable solution at NIR wavelengths. Additionally, other nanomaterials such as TCO and TMO could potentially allow the viability of the nanocube nanostructure, but since lithographic fabrication methodologies with these nanomaterials is not very frequent to find, a near future implementation cannot be foreseen.

Nanorods are a very versatile nanostructure, both in terms of fabrication, LSPR band tuning and spectral characteristics, finding several uses in the fields of chemical and biological sensing [157]. Spectrally, they show two resonant modes, namely a transversal mode, associated with oscillations along their width, and a longitudinal mode, caused by oscillations along their length. As we move towards higher wavelengths, namely aiming for sensing around 1550 nm, the longitudinal mode is the one of interest, namely due to its higher wavelength position, increased refractometric sensitivity and resonance strength. Geometrically the aspect ratio was found to be the most common and effective tunning parameter, with a linear relationship between 95 and 115 nm/aspect ratio, as reported elsewhere [60,61,62,159,160]. In comparison to the NS case, where was presented a tuning parameter on NS diameter of 4.7 nm/nm (wavelength/diameter), the NR can have a largely more effective tuning mechanism. Namely for aspect ratios as low as 10 will place the longitudinal band of a gold NR at 1500 nm. Therefore, regarding wavelength tuning this nanostructure possess the best characteristics of the prior ones. Regarding fabrication, NRs are also very versatile, with the possibility of synthesis via colloidal methods, as well as fabrication via lithographic means. This presents an enormous advantage since it could potentially be chosen a colloidal protocol that will produce a large amount of Au NRs in a quick manner and with high geometrical control. The refractometric characteristics of these NRs also highly present performant nanostructures with RI sensitivities around 473 nm/RIU at 1064 nm, with a sensitivity dependence on wavelength of 0.66 nm/RIU/nm (RI sensitivity/wavelength). Therefore, predicting refractometric sensitivities on the order of 990 nm at 1500 nm. This places the NRs as the most prominent and mature way of devising NIR LPRS based biosensors with ease.

Nanorings are a very interesting nanostructure regarding their optical characteristic’s dependency on geometrical factors. The ring thickness to diameter ratio being the most relevant one to tune the LSPR band. It has been reported that in complex nanostructures, such as sphere-in ring, the resonance band can be tuned from the VIS to NIR region by adjusting the thickness and cavity sizes [180]. Their fabrication is achieved mainly through lithographic means, and in special cases with NSL. Colloidal mechanisms offering good dimensional control to fabricate such nanostructures were not found to be reported. Thus, in comparison to NRs they are not so easily obtained. Nevertheless, their spectral features are among the best that can be obtained with isolated NPs. There are reports of highly performant LSPR sensors. Larsson et al. [23] reports such example with a LSPR band at 1350 nm, with a Q-factor of 3.4 and incredibly high RI sensitivities of 880 nm/RIU. This value surpasses any other nanostructures of the same nanomaterial. Toma et al. [75] presented another relevant work with Au nanorings, achieving LSPR bands from 1050 to 1650 nm, with Q-factors greater than 4 and sensitivities between 805 and 1020 nm/RIU. Indubitably the highest refractive sensitivity reported among the nanostructures.

Nanopillars are nanostructures that can benefit from a hybrid plasmonic mechanism, benefiting from both SPR and LSPR phenomena, since they are generally so closely packed or have a conductive layer at their basis that plasmonic coupling occurs and SPR modes can propagate. The most common geometrical parameters to tune the LSPR bands are the height, radius, and inter-pillar distance. These parameters can be easily adjusted to support operation at the 1500 nm band since typically these nanostructures are fabricated thru lithographic means. Works such as that presented by Etin et al. [184] have shown high RI sensitivities of 675 nm/RIU with such nanostructures operating at a wavelength of 950 nm.

Nanoshells are characterized by a dielectric core and a metallic shell, typically Au and SiO_2_. These nanostructures were reported to be capable of being tuned wavelengths up to 2000 nm, thru the increase of the core-shell ratio, as shown in several works [187,188]. Unfortunately, experimental reports have not produced such wavelengths, with the maximum values being reported by Brinson et al. [189] with LSPR bands at 1200 nm and Xie et al. [190] at 1320 nm with these nanoshells. Moreover, their RI sensitivity are also not quite on pair with the other discussed nanostructures, presenting 270 nm/RIU at wavelengths of 900 nm.

Nanocrescents are a kind of nanostructure typically produced via NSL lithography. An interesting characteristic is related to their refractometric sensitivity, which take advantage of Fano resonances originated at their tips. As so, a very precise control of their geometries is required for them to perform as expected. Works such as reported by Jiang et al. [196] shown RI sensitivities of 332 nm/RIU in a wavelength range of 1200 nm to 2000 nm, therefore an already viable nanostructure but presenting less sensitivity when compared with the more easy to produce NRs. Nevertheless, it could pose as an interesting alternative as a nanostructure for NIR in the future, even though it still needs development over geometry control and RI sensitivity.

Striding away from single and isolated NPs, when those are in considerably proximity, interparticle coupling occurs, changing the plasmonic resonance conditions. Among such nanostructures nanodisks arrays, typically fabricated through lithography methods, are of special interest. The nanodisk main tuning parameters present linear relationships between diameter, period, and thickness to the LSPR wavelength on a ratio of 0.3 nm/nm (wavelength/diameter), 1.4 nm/nm (wavelength/Period) and −3.2 nm/nm (Wavelength/thickness), respectively. A comparison must be made with NRs aspect ratio, because nanodisk arrays require greater physical changes to achieve large tunability, even using the three parameters, when compared to the near 100nm/aspect ratio for the NRs. Regarding RI sensitivities, the magnitudes obtained with these nanostructures surpass what can be obtained with nanocubes or NS, but not NRs or nanorings, with sensitivities of 219 nm/RIU at 741 nm and 345 nm/RIU at 1171 nm. Nevertheless, active developments on this nanostructure have been reported even at the MIR by Chang et al. [71], employing a MIM configuration. This work operated around 5000 nm with a RI sensitivity of 1450 nm/RIU, which agrees well with the proportional constant of 0.292 nm/RIU/nm, giving only a difference of a mere 20 nm/RIU. Finally, biosensing applications at NIR wavelengths have also been reported for such nanostructures, as the work presented by Jiang et al. [209], at wavelengths up to 1025 nm measuring streptavidin, BSA, and Ig antibodies.

Nanotriangle arrays have also been reported with several nanomaterials, showing interesting tuning parameters proportions. Namely, Jensen et al. [211] reported Ag nanotriangles with a proportionality factor of 4 nm/nm (wavelength/thickness), while Liang et al. [212] reported a proportionality factor of 13.5 nm/nm (wavelength/thickness) with VO2 nanotriangles. This more than tripled that tuning parameter and shows how different nanomaterials affect each nanostructure optical and refractometric properties.

Nanohole arrays also present interesting optical properties, as shown by Zhao et al. [216] with a gold nanohole array showing RI sensitivities of 487 nm/RIU at 800 nm, while presenting a method to transfer the nanohole array to the fiber by a templated transfer method with a UV curing adhesive. It is important to notice that, due to the lithography techniques involved, the RI sensitivity gain and LSPR wavelength tunability requires very precise and therefore costly mechanisms to produce such nanostructures. Thus, in general, making them not very suitable for wide use.

Finally, superstructures may present interesting optical characteristics and a new method to achieve LSPR sensing at NIR wavelengths, through size and shape modifications. A major difference between this and other nanostructures lies with the fact that superstructures, since they are an ensemble of simpler nanostructures, both spectral and refractometric properties are not well defined, as the case of aggregation phenomena which can produced similar chaotic behavior. As so, since in this review, we are concerned with obtaining nanostructures targeting high performant sensing at higher wavelengths, the plasmonic band tuning is of paramount relevance. Finally, transfer mechanisms to optical fibers are also a problem to address, and the use of these superstructures will create additional challenges.

**Figure 18:**
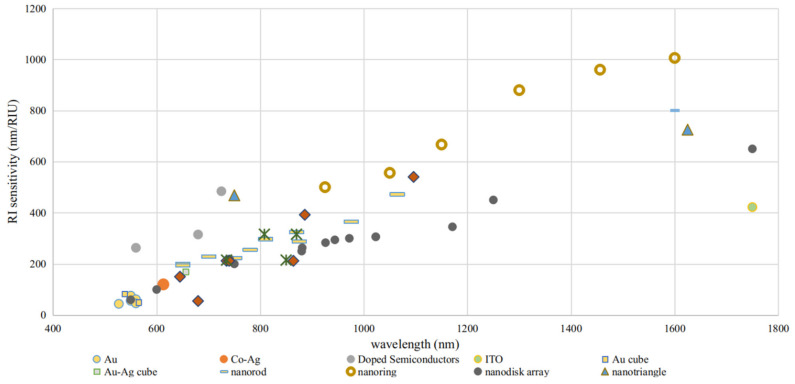
Sensitivity vs. wavelength comparison for the most performant nanostructures towards NIR sensing.

As was seen there is an immense variety of nanostructures, so graphical comparison between them in terms of LSPR wavelength and RI sensitivity can prove to be beneficial. This is presented in Figure 18.

As was stated, the NRs are a great candidate for the intended applications. However, the lack of reports on RI sensitivity of Au nanorods with aspect ratios greater than 8, causes them not to be presented as the best candidate, nevertheless there is not a physical or synthesis limitation that could present a barrier to their usage in the wavelength range of interest. Moreover, nanorings are shown as good nanostructural candidates presenting the highest refractometric sensitivities.

Stepping back the beginning of the nanostructure discussion, with the NS, the gold NS are considered the worst nanostructure for the intended application. Nevertheless, employing a TCO nanomaterial configuration completely shifted not only the resonance band towards the NIR-MIR range, but it also enhanced the refractometric capabilities by an order of magnitude. Therefore, it is shown that nanomaterials could effectively be a solution in specific cases, and one should not restrict themself to the use of typical metals.

In the figure, it is also possible to see that nanobipyramids perform very similarly to NRs and nanorice datapoints at 1600 nm, which can be seen to intercept the tendency for the NRs if a linear regression was also drawn in the figure, which we did not do, to avoid overpopulating the graphic.

In summary, there are several approaches that enable the LSPR at NIR wavelengths. The most suitable solution depends on the application and resources available for synthesis. One can achieve NIR LSPR with Au NR with aspect ratios of 10 that can be synthesized using colloidal methods, using Sn doped ITO NP produced via spin-casting or ultra-sensitive arrays produced by lithographic means.

Moreover, the research for new materials and nanostructure optimization towards the NIR, where low-cost and long-range sensing capacity can become a reality, when supported by optical fiber technology, presents itself as a promising field to be explored soon. This will provide a step towards the affirmation of real world and large-scale implementation of plasmonic sensing devices out of laboratory environments.

## Data Availability

Not applicable.
